# Brf1 loss and not overexpression disrupts tissues homeostasis in the intestine, liver and pancreas

**DOI:** 10.1038/s41418-019-0316-7

**Published:** 2019-03-11

**Authors:** Dritan Liko, Louise Mitchell, Kirsteen J. Campbell, Rachel A. Ridgway, Carolyn Jones, Kate Dudek, Ayala King, Sheila Bryson, David Stevenson, Karen Blyth, Douglas Strathdee, Jennifer P. Morton, Thomas G. Bird, John R. P. Knight, Anne E. Willis, Owen J. Sansom

**Affiliations:** 1 CRUK Beatson Institute, Garscube Estate, Switchback Road, Glasgow, G61 1BD UK; 2 MRC Toxicology Unit, Hodgkin Building Lancaster Road, Leicester, LE1 9HN UK; 3 Institute of Cancer Sciences, University of Glasgow, Glasgow, G61 1BD UK

**Keywords:** Oncogenes, Cancer models

## Abstract

RNA polymerase III (Pol-III) transcribes tRNAs and other small RNAs essential for protein synthesis and cell growth. Pol-III is deregulated during carcinogenesis; however, its role in vivo has not been studied. To address this issue, we manipulated levels of Brf1, a Pol-III transcription factor that is essential for recruitment of Pol-III holoenzyme at tRNA genes in vivo. Knockout of *Brf1* led to embryonic lethality at blastocyst stage. In contrast, heterozygous *Brf1* mice were viable, fertile and of a normal size. Conditional deletion of *Brf1* in gastrointestinal epithelial tissues, intestine, liver and pancreas, was incompatible with organ homeostasis. Deletion of *Brf1* in adult intestine and liver induced apoptosis. However, *Brf1* heterozygosity neither had gross effects in these epithelia nor did it modify tumorigenesis in the intestine or pancreas. Overexpression of BRF1 rescued the phenotypes of *Brf1* deletion in intestine and liver but was unable to initiate tumorigenesis. Thus, Brf1 and Pol-III activity are absolutely essential for normal homeostasis during development and in adult epithelia. However, Brf1 overexpression or heterozygosity are unable to modify tumorigenesis, suggesting a permissive, but not driving role for Brf1 in the development of epithelial cancers of the pancreas and gut.

## Introduction

RNA polymerase III (Pol-III) transcribes tRNAs and other short non-coding RNAs that are important in protein production. Pol-III is the largest of the RNA polymerases containing 17 subunits, all necessary for transcription and cell viability [1–4]. The recently solved structure of initiating Pol-III reveals its similarity to Pol-II, and demonstrates the multitude of interactions required for transcription initiation [5, 6]. Pol-III holoenzyme is directed to the majority of its target genes via two Pol-III-associated transcription factor complexes, TFIIIB and TFIIIC [7, 8]. TFIIIC recognises sequences in the body of Pol-III transcribed genes and recruits TFIIIB [4, 8], which in turn recruits Pol-III in order to commence transcription [9–12]. Recruitment of TFIIIB is the rate-limiting step in Pol-III-dependent transcription [9]. TFIIIB is composed of three proteins BDP1, TBP and BRF1. While TBP is shared between all three polymerases, BDP1 and BRF1 are exclusively utilized by Pol-III [8]. The BRF1 homologue BRF2 forms a distinct complex with BDP1 and TBP to promote transcription of type III Pol-III targets [13, 14]. The BDP1/TBP/BRF1 complex is required for type I and II target genes, which accounts for the majority of Pol-III transcription. Growth related kinases, oncoproteins and tumor suppressors regulate Pol-III transcription, assuring appropriate levels of Pol-III transcripts [15, 16]. The majority of these signals are channelled through BRF1, making it a signal hub at tRNA genes [17–25].

Pol-III transcription is vital for cellular maintenance and important for growth and proliferation. For example, Pol-III transcription is upregulated during cardiomyocyte proliferation and drops during cell differentiation [26, 27]. Pol-III transcription is essential in organismal homeostasis and development. In zebrafish, a 60% reduction in Pol-III activity had a profound effect during larvae development, especially in the intestine and exocrine pancreas [28]. Moreover, deletion of *Brf1* in *Drosophila* reduced pupa size and gave rise to smaller adult flies [29, 30].

In mice, deletion of selenocysteine-tRNA causes a pre-implantation defect [31]. Furthermore homozygous deletion of *La* protein, a known positive regulator of Pol-III transcription, blocks embryo development pre-implantation [32, 33]. However, *La* proteins affect other pathways besides Pol-III transcription [34]. During mouse embryo development the pre-implantation stage is characterised by a burst of transcription and translation that may be dependent on Pol-III activity [35, 36].

Pol-III levels positively correlate with cellular transformation [37–39] and subunits of Pol-III are known to be overexpressed in tumours [16, 22, 40]. Data also suggest that cancer cell lines regulate tRNA availability in order to support cell growth and proliferation [40]. For example, tRNA^iMET^, the initiator tRNA, is overexpressed drives cancer progression [40, 41]. Since Brf1 is essential for recruitment of Pol-III holoenzyme at tRNA genes it is unsurprising that knockdown of BRF1 protects against transformation in transformed cell lines [42]. Moreover, BRF1 protein may serve as a biomarker in hepatocellular carcinoma where levels of BRF1 were higher and correlated with poor survival [43]. Importantly, elevated levels of tRNA^iMET^ drive tumor cell migration without affecting proliferation [44, 45]. Taken together these data suggest that the link between Pol-III function and tumorigenesis is not necessarily direct and requires further investigation.

To address this we modulated Brf1 levels in mice finding that genetic ablation of *Brf1* stops embryonic development and is incompatible with adult organ homeostasis. Adult epithelial cells lacking Brf1 show a reduction in tRNAs and polysomes, followed by p53 induction and apoptosis. In contrast, heterozygous loss or overexpression of *Brf1* does not alter homeostasis or tumorigenesis. Taken together these studies suggest a crucial function for Pol-III activity in both normal and cancer cells, but does not limit tumour initiation in the intestine and pancreas.

## Results

### BRF1 deletion is embryonically lethal

**Fig. 1:**
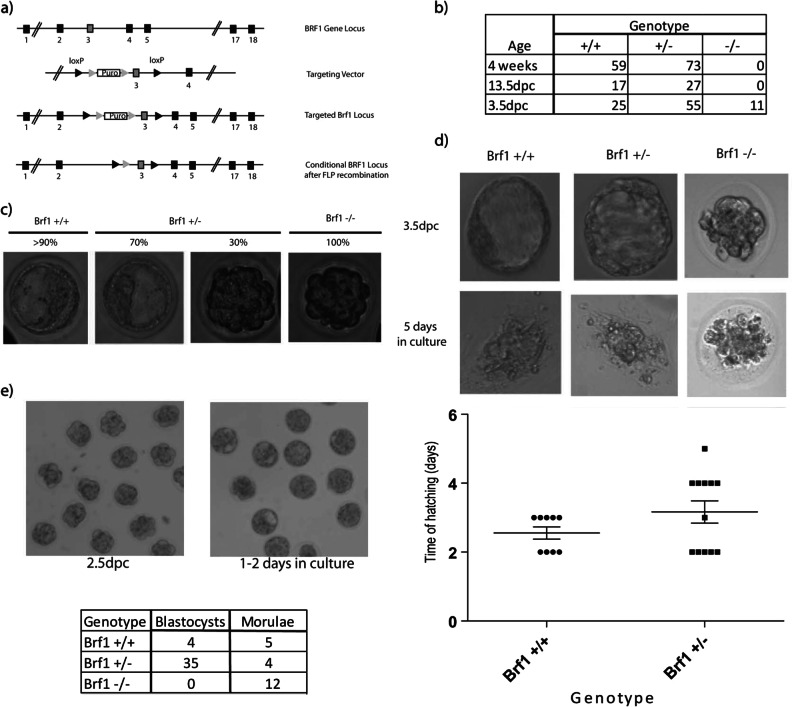
Brf1 deletion causes a pre-implantation defect. **a** Strategy used to generate the *Brf1*^*flox*^ allele. Grey arrowheads are *Flip* sites used to excise the puro cassette; black arrowheads are *loxP* sites inserted flanking exon 3 of the *Brf1* locus. **b** Table of mice and embryos generated after crossing *Brf1*^*+/*−^ mice (dpc, days post coitus). **c** Morphology of 3.5dpc embryos captured with a light microscope. Genotypes are as shown. Percentages depict number of embryos at the blastocyst stage, characterised by vacuole like presence, or the morulae stage. **d** Top panels show pictures of embryos at 3.5dpc when growth in vitro commenced and after 5 days in culture. *Brf1*^*+/+*^ and *Brf1*^*+/*−^ embryos shown after 5 days have colonised the plate. Bottom panel shows graphical representation of the time embryos hatch and rupture the zona pellucida for *Brf1*^*+/+*^ (*n* = 9) and *Brf1*^*+/*−^ (*n* = 12) embryos. **e** Top panels show 2.5dpc embryos in vitro at the start of culture and after 2 days in culture. The table shows the genotypes of individual embryos harvested after 2 days in culture

**Fig. 2:**
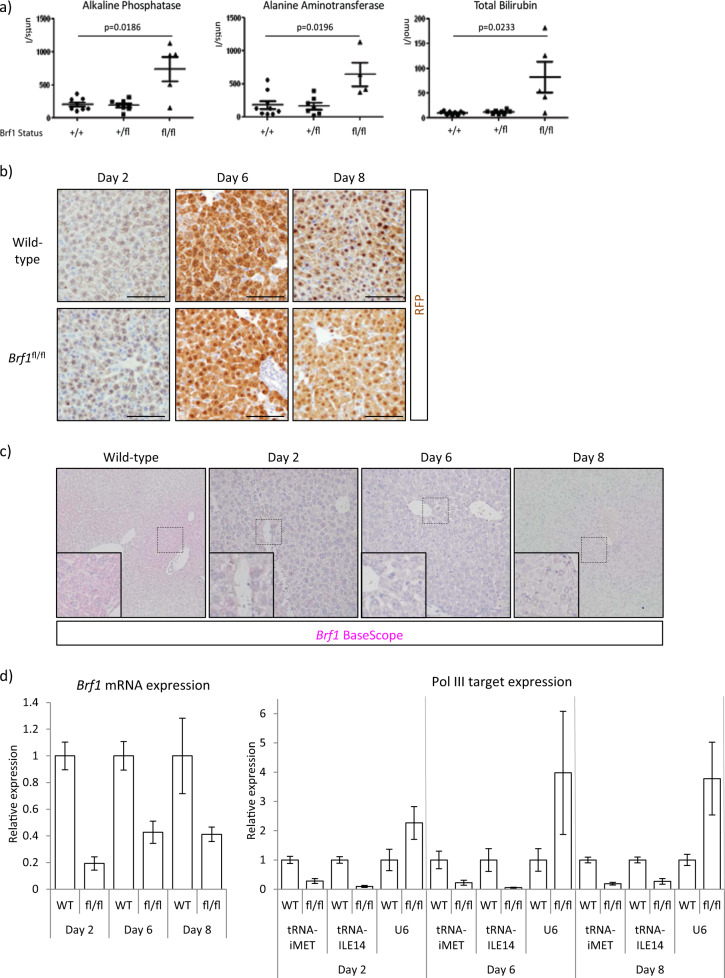
BRF1 down-regulation in the livers of *AhCre Brf1*^*fl/fl*^ mice. **a** Graphs showing levels of the specified liver enzymes in the blood of mice of the indicated genotypes. *Brf1*^+/+^ mice are a combination of *AhCRE Brf1*^+/+^ and *AhCre* negative mice, *Brf1*^+/fl^ mice are *AhCRE Brf1*^+/fl^ mice, whilst *Brf1*^fl/fl^ mice are *AhCre Brf1*^fl/fl^ mice. *P*-values are calculated using a Mann–Whitney non-parametric test and show the significance between +/+ and fl/fl values. **b** Immunohistochemistry for RFP performed on liver sections. *Brf1*^+/+^ mice are a combination of *AhCRE Brf1*^+/+^ and *AhCre* negative mice, whilst *Brf1*^fl/fl^ mice are *AhCre Brf1*^fl/fl^ mice. Day post-induction with β-naphthoflavone is indicated above the panels. **c** BaseScope hybridisation showing expression of *Brf1* mRNA in wild-type mice and *Brf1*^fl/fl^ mice at 2, 6 and 8dpi. **d** Graphs showing reduction of *Brf1* mRNA levels and Pol-III target levels upon *Brf1* loss. Graph on the left depicts levels of *Brf1* mRNA measured by qPCR. Reduction in *Brf1* mRNA levels is significant for each day (*p* < 0.05). Graph on the right depicts levels of two tRNAs, tRNA^iMET^ and tRNA^ILE14^, and the U6 RNA. Changes in tRNA levels are significant on each day (*p* < 0.05). Mouse genotype and day post-induction is shown on the x-axis. Values are plotted relative to WT control for each day shown

To assess the effect of *Brf1* deletion in mice exon 3 was flanked by two LoxP sites creating a conditional *Brf1* allele (Fig. 1a and S Fig. 1). We generated *Brf1* heterozygous animals (*Brf1*^+/−^) by crossing mice containing the conditional *Brf1* allele (*Brf1*^fl/+^) to deleter-Cre mice (S Fig. 2A) [46]. *Brf1*^*+/*−^ mice, heterozygous for Brf1, were comparable to wild-type controls in terms of body weight and other visible phenotypes (S Fig. 2B). No *Brf1*^−/−^ mice were obtained upon inter-breeding of *Brf1*^*+/*−^ mice, suggesting that *Brf1* deletion is embryonically lethal (Fig. 1b). Furthermore, the ratio of heterozygous to wild-type mice was significantly skewed from an expected Mendelian ratio of 1:2:1 (Chi-square test, *p* < 1 × 10^−4^ for mice at 4 weeks), suggesting an effect of Brf1 heterozygosity during development.

### BRF1 is essential for blastocyst formation

Embryos undergo three rounds of cell division to reach the “8-cell” stage, then progresses to morulae and undergo further division and differentiation to give rise to blastocysts that implants into the uterine wall. During implantation a dramatic increase in growth and energy usage is observed [47]. Harvesting embryos at either 3.5 days post coitus (dpc) or 13.5dpc revealed the presence of *Brf1*^−/−^ embryos only at 3.5dpc, suggesting that *Brf1* is essential for embryo development after 3.5dpc (Fig. 1b). At 3.5dpc, >90% of Brf1^+/+^ mouse embryos were at the blastocyst stage, compared to only 70% of *Brf1*^+/−^ embryos and none of the *Brf1*^−/−^ embryos (Fig. 1c). These results suggest an essential role for *Brf1* during blastocyst formation at 3.5dpc. It is worth noting that *Brf1* heterozygosity may repress the passage from morulae to blastocyst (*p* < 1 × 10^−4^, *n* = 11), in line with reduced Brf1 perturbing early embryonic development.

To distinguish if loss of *Brf1* causes a delay or a complete block of the passage from morulae to blastocyst we isolated 3.5dpc embryos from *Brf1*^+/−^ inter-crosses and cultured them in vitro. After 5 days in culture a considerable number of embryos hatched and colonised in vitro, mimicking uterine implantation (Fig. 1d). No *Brf1*^−*/*−^ cultured embryos advanced to blastocyst stage, whereas all *Brf1*^*+/+*^ embryos hatched at 2 or 3 days after plating (5.5dpc and 6.5dpc). A number of *Brf1*^*+/*−^ embryos hatched at later time points, underscoring the role of Brf1 during embryo development (Fig. 1d).

Eight-cell embryos were collected at 2.5dpc and cultured for 2 days to monitor progression to blastocyst. While wild-type and *Brf1*^*+/*−^ 8-cell embryos became blastocysts within 2 days, none of the 12 *Brf1*^−*/*−^ embryos isolated progressed (Fig. 1e). Taken together these data show that Brf1 is essential for blastocyst formation and that *Brf1* heterozygosity slows progression, which may account for sub-Mendelian ratios of pups.

### BRF1 is essential for liver function

We next assessed the impact of attenuating specific Pol-III activities in adult mice using *AhCre*. This Cre recombinase is under the control of a *Cyp1a1* promoter that can be induced by β-naphthoflavone [48]. Recombination occurs in hepatocytes and the enterocytes of the small intestine [48], allowing the comparison of slowly dividing hepatocytes with highly proliferative intestinal enterocytes and stem cells.

At 8 days post-induction (dpi) we observed increased levels of circulating Bilirubin, alkaline phosphatase and alanine aminotransferase in *AhCre Brf1*^*fl/fl*^ mice (Fig. 2a) consistent with liver injury and dysfunction. Lineage tracing experiments with the *Rosa26*^*LSL-RFP*^ reporter allele showed that virtually all hepatocytes retained RFP expression (Fig. 2b). Furthermore, *Brf1* mRNA levels were reduced as visualised by BaseScope and quantified by qPCR (Fig. 2c, d). Importantly this resulted in a specific loss of Brf1 function, as tRNA^iMET^ and tRNA^ILE14^ levels were reduced within 2 days of induction and remained low at 6dpi and 8dpi, whereas expression of the Brf1-independent Pol-III target U6 was not reduced (Fig. 2d).

**Fig. 3:**
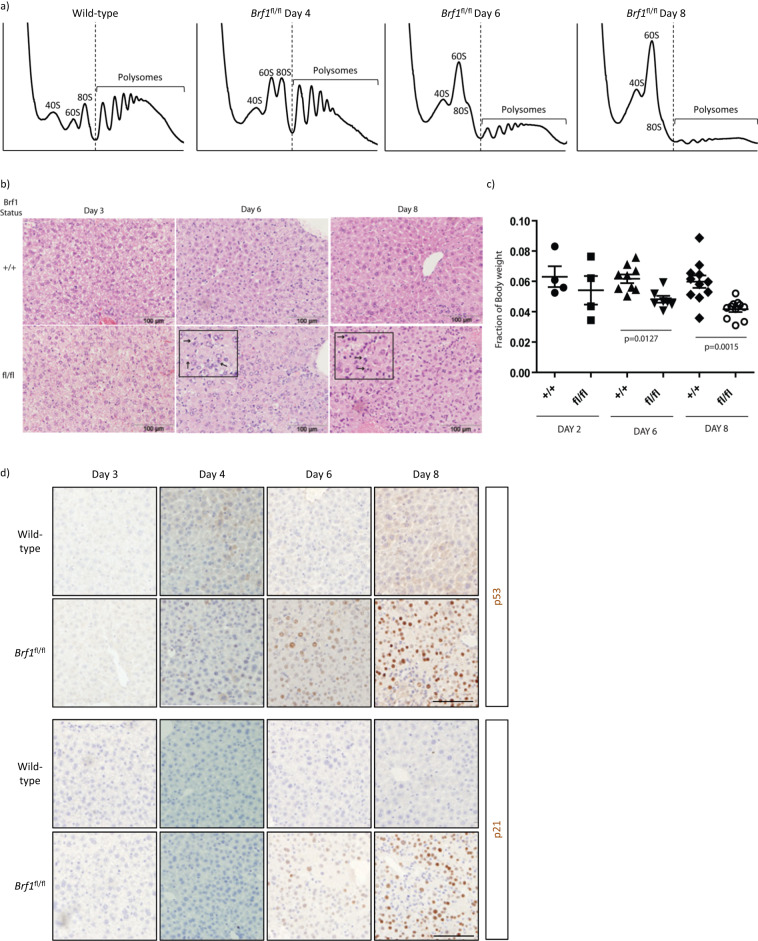
Brf1 is essential for adult mouse liver function. **a** Polysome profiles from liver samples of *Brf1*^+/+^ mice (Ah-Cre *Brf1*^+/+^) harvested at day 8 post induction and *Brf1*^fl/fl^ (Ah-Cre *Brf1*^−/−^) mice harvested at days 4 (*n* = 2), 6 (*n* = 3) and 8 (*n* = 4) post induction as shown. *Brf1*^+/+^ mice are a combination of Ah-CRE *Brf1*^+/+^ and mice without Ah-Cre (*n* = 3). **b** H&E stained mouse liver samples. *Brf1*^+/+^ mice are a combination of *AhCRE Brf1*^+/+^ and *AhCre* negative mice, whilst *Brf1*^fl/fl^ mice are *AhCre Brf1*^fl/fl^ mice. Day post-induction with β-naphthoflavone is indicated above the panels. Insets for day 6 and day 8 samples are 400x magnifications to better show tissue morphology. Arrows in the inserts show immune cell infiltration and collapsing hepatocytes. **c** Graph depicting mouse liver weight as a fraction of total body weight. Genotypes and day post-induction are shown on the *x*-axis. *Brf1*^+/+^ mice are a combination of *AhCRE Brf1*^+/+^ and *AhCre* negative mice, whilst *Brf1*^fl/fl^ mice are *AhCre Brf1*^fl/fl^ mice. *P*-values were calculated using a Mann–Whitney test to compare +/+ and fl/fl data. Changes at day 2 are not significant. Between 4 and 10 mice were analysed per genotype per time point. **d** IHC for p53 or p21, as indicated, on liver samples from mice harvested at 3, 4, 6 and 8dpi. Day post-induction with β-naphthoflavone is indicated above the panels. *Brf1*^+/+^ mice are a combination of *AhCRE Brf1*^+/+^ and *AhCre* negative mice, whilst *Brf1*^fl/fl^ mice are *AhCre Brf1*^fl/fl^ mice

Reduced tRNA levels are likely to decrease protein synthesis. To investigate this we quantified the translation activity in *Brf1* deficient hepatocytes using sucrose density gradient analysis at 4, 6 and 8dpi. Livers at 4dpi showed a decrease in polysome loading that decreased further during the time course (Fig. 3a). Indeed, at 8dpi there are virtually no polysomes remaining, consistent with a shortage of tRNAs dramatically decreasing translation rates.

Consistently, *Brf1* deletion was detrimental for liver homeostasis, with morphology notably altered 6 and 8dpi. *Brf1* deficient livers showed high levels of apoptosis, immune cell infiltration and necrosis (Fig. 3b). Moreover, *Brf1* deficient livers were significantly smaller at day 8 (Fig. 3c). *Brf1* deletion resulted in a modest induction of p53 at 4dpi, which increased further on days 6 and 8 (Fig. 3d). The stress markers p21 and γH2AX were also increased, but only from 6dpi, with prominent induction at day 8 (Fig. 3d, S Fig. 3A). Some hepatocytes also stained for cleaved caspase 3, a feature of apoptosis, and cytoplasmic cytokeratin, a feature of collapse (S Fig. 3B). Importantly, the liver stress phenotype occurs after the molecular effect of *Brf1* deletion on translation seen at day 4, implicating suppressed translation as the driver of liver damage.

**Fig. 4:**
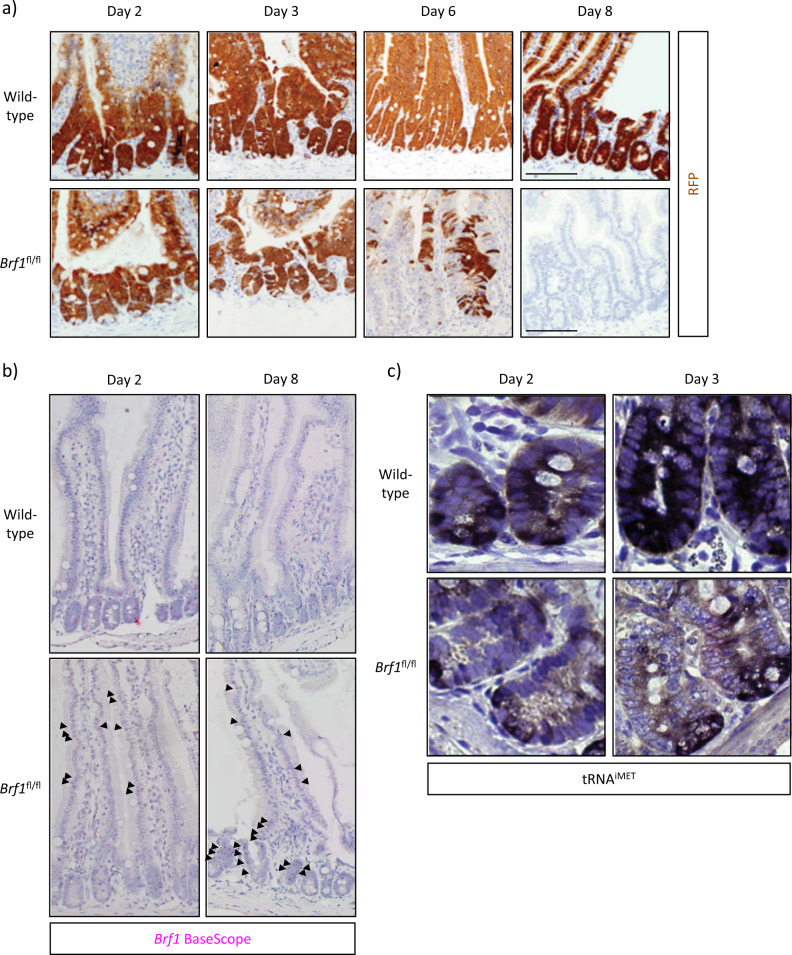
Loss of recombined cells upon *Brf1* deletion in intestinal crypts. **a** Immunohistochemistry for RFP on sections of FFPE intestinal tissue. *Brf1*^+/+^ mice are a combination of *AhCRE Brf1*^+/+^ and *AhCre* negative mice, whilst *Brf1*^fl/fl^ mice are *AhCre Brf1*^fl/fl^ mice. Day post-induction with β-naphthoflavone is indicated above the panels. Scale bar in 100 µm. **b** BaseScope hybridisation for *Brf1* mRNA expression in the intestines of wild-type (induced *AhCre* negative *Brf1*^*fl/fl*^ mice) or *Brf1*^*fl/fl*^ mice at 2 and 8dpi. **c** In situ hybridisation using a probe against tRNA^iMET^. *Brf1*^+/+^ mice are a combination of *AhCRE Brf1*^+/+^ and *AhCre* negative mice, whilst *Brf1*^fl/fl^ mice are *AhCre Brf1*^fl/fl^ mice. Day post-induction with β-naphthoflavone is indicated above the panels

To remove any confounding effects of extrahepatic recombination of the *Brf1* allele we used a liver tropic adeno-associated virus construct (AAV8) to deleted *Brf1* only in the liver. The rapid and dramatic phenotype of *Brf1* deletion was recapitulated in this system, where within 4 days of AAV treatment p53 levels were induced and within 6 days p21 and cleaved caspase 3 levels were upregulated in *Brf1*^*fl/fl*^ mice (S Fig. 4A). AAV8-CRE treatment brought upon a liver collapse phenotype in *Brf1*^*fl/fl*^ mice as characterised by increased pan-cytokeratin staining and circulating levels of bilirubin, alkaline phosphatase and alanine aminotransferase (S Fig. 4B). This was accompanied by reduced liver to body weight in (S Fig. 4C) and decreased tRNA^ILE14^ but no reduction in U6 RNA expression (S Fig. 4D).

Importantly, heterozygous deletion of *Brf1* using AAV8-CRE did not result in a reduction of Brf1 protein, despite efficient loss of the protein following homozygous deletion (S Fig. 4E). This indicates that heterozygous deletion of the Brf1 gene is not sufficient to suppress protein expression, consistent with no effect on Brf1-dependent Pol-III transcription (S Fig. 4D).

### BRF1 is essential for gut homeostasis

**Fig. 5:**
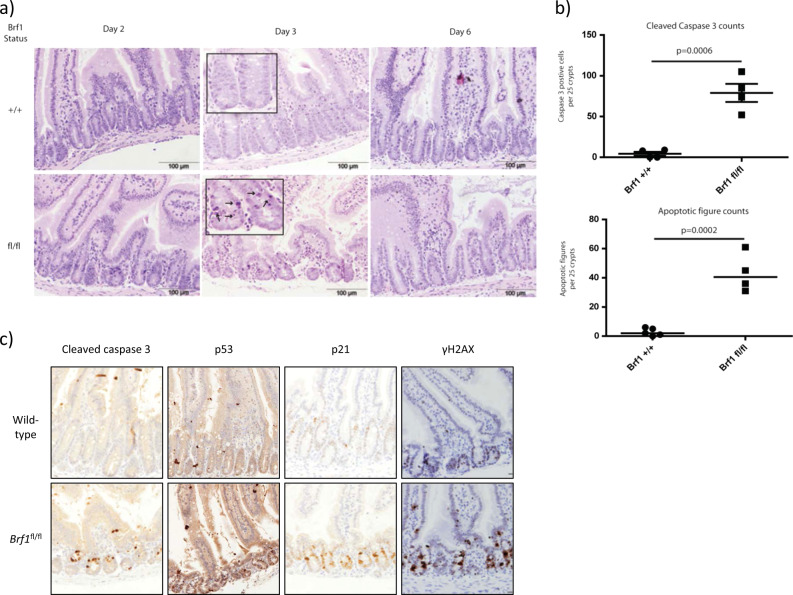
Brf1 is essential in mouse gut homeostasis. **a** H&E stained sections of mouse intestinal tissue. *Brf1*^+/+^ mice are a combination of *AhCRE Brf1*^+/+^ and *AhCre* negative mice, whilst *Brf1*^fl/fl^ mice are *AhCre Brf1*^fl/fl^ mice. Day post-induction with β-naphthoflavone is indicated above the panels. Insets of day 3 samples are 400x magnification to better show tissue morphology. Arrows in the insets show immune cell infiltration and cells undergoing apoptosis. **b** Top graph shows quantification of cleaved caspase 3 counts. Genotypes are shown on the *x*-axis and counts per 25 full crypts are shown on the y-axis. Bottom graph shows quantification of apoptotic figures. Genotypes are shown on the *x*-axis and apoptotic figure counts per 25 full crypts are shown on the *y*-axis. *P*-values calculated for a Mann–Whitney non-parametric test are shown. Four mice were used for each genotype. **c** IHC on sections of intestine from mice harvested 3dpi using the antibodies indicated. *Brf1*^+/+^ mice are a combination of *AhCRE Brf1*^+/+^ and *AhCre* negative mice, whilst *Brf1*^fl/fl^ mice are *AhCre Brf1*^fl/fl^ mice

We next examined intestinal homeostasis in *AhCre Brf1*^*+/+*^, *Brf1*^*fl/+*^ and *Brf1*^*fl/fl*^ mice from 2 to 8dpi. *Rosa*^*LSL-RFP*^ reporter recombination was observed within 2dpi in line with reduced *Brf1* mRNA expression (Fig. 4b). Consistent with reduced Brf1 function there was a reduction in Pol-III transcript levels by in situ hybridisation against tRNA^iMET^ at both 2 and 3dpi (Fig. 4a). Therefore, we conclude that from 2dpi, Brf1, and specific Pol-III activity is reduced in the intestinal epithelium. Histologically we observed smaller, collapsing crypts and induction of cleaved caspase 3 (Fig. 5a, b). Moreover, at 3dpi, there was an increase in staining for p53, γH2AX and p21 (Fig. 5c). Therefore, similar to the liver, loss of Brf1 induces activation of p53, p21 and γH2AX and apoptosis.

The intestinal epithelium has a remarkable ability to regenerate, with previous studies showing that deletion of genes required for viability leading to regeneration of the intestine from non-recombined cells [48–50]. By 8dpi the histology of the intestinal epithelium appeared normal. In contrast to day 2, when *Brf1* mRNA expression was reduced and RFP expression was high, at 8dpi we saw high *Brf1* expression and loss of RFP expression (Fig. 4a). This strongly suggests that at later time points Brf1 deficient cells are lost, being replaced by non-recombined Brf1 expressing cells.

### BRF1 overexpression rescues loss of BRF1 but does not induce proliferation

**Fig. 6:**
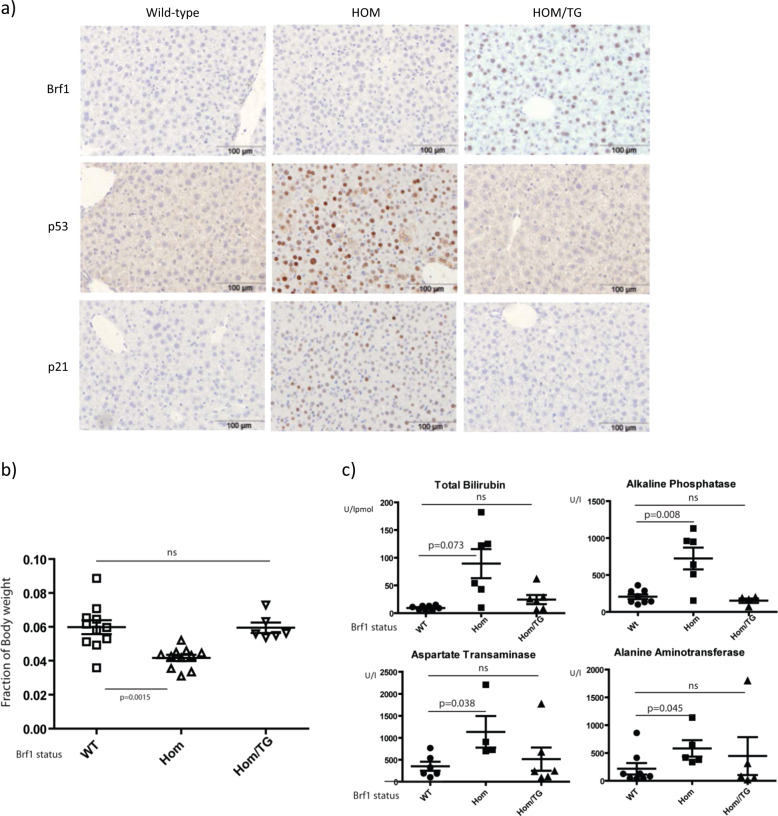
Expression of human BRF1 rescues loss of *Brf1* liver phenotype. **a** IHC for BRF1, p53 and p21 on liver samples from mice harvested at day 8 post-induction. Mouse genotypes are indicated above the panels. WT mice are a combination of *AhCre Brf1*^*+/+*^ mice and *AhCre* negative mice, Hom mice are *AhCre Brf1*^*fl/fl*^ and Hom TG mice are *AhCre Brf1*^*fl/fl*^ *Hprt*^*LSLBRF1*^ mice containing an extra copy of human *BRF1*. **b** Graph depicting mouse liver weight as a fraction of total body weight of animal harvested 8dpi. Genotypes are indicated on the *x*-axis. WT mice are a combination of *AhCre Brf1*^*+/+*^ mice and *AhCre* negative mice (*n* = 11), Hom mice are *AhCre Brf1*^*fl/fl*^ (*n* = 11) and Hom TG mice are *AhCre Brf1*^*fl/fl*^ *Hprt*^*LSLBRF1*^ mice containing an extra copy of human *BRF1* (*n* = 6). *P*-values are calculated for a non-parametric Mann–Whitney test. Changes between WT and Hom/TG mice are not significant whereas changes between *Brf1*^*fl/fl*^ and WT animals are significant (*p* < 0.05). **c** Graphs showing levels of the specified liver enzymes in the blood of mice with the indicated genotypes. WT mice are a combination of *AhCre Brf1*^*+/+*^ mice and *AhCre* negative mice (*n* = 8), Hom mice are *AhCre Brf1*^*fl/fl*^ (*n* = 5) and Hom TG mice are *AhCre Brf1*^*fl/fl*^ *Hprt*^*LSLBRF1*^ mice containing an extra copy of human *BRF1* (*n* = 5). *P*-values are calculated for a non-parametric Mann–Whitney test. Changes between WT and Hom/TG mice are not significant

We next assessed the consequences of increased expression of Brf1. The liver is slowly proliferative, making this an ideal organ to assess if Brf1 induction promotes proliferation and growth. A lox-STOP-lox allele containing human *BRF1* was engineered to express from the *Hprt* locus upon Cre induction (*Hprt*^*LSL-BRF1*^). To validate the *BRF1* transgene, we assessed whether its expression rescues the phenotype of Brf1 loss. We generated *AhCre Brf1*^*fl/fl*^ *Hprt*^*LSLBRF1*^ mice (HOM/TG) where, upon induction, both endogenous copies of *Brf1* are deleted and human *BRF1* is expressed. Livers from these mice were harvested 8dpi and compared with those from *AhCre Brf1*^*+/+*^ (WT) and *AhCre Brf1*^*fl/fl*^ (HOM) mice. Human BRF1 was detected by immunohistochemistry in the HOM/TG mice, indicating expression from the transgene (Fig. 6a) and *Brf1* mRNA expression was increased in these mice (S Fig. 5A). Furthermore, the levels of tRNA^iMet^ and tRNA^ILE14^ are also higher in the HOM/TG mice compared with HOM mice (S Fig. 5B) demonstrating that human BRF1 is able to restore, at least in part, Pol-III function following loss of endogenous *Brf1*. Importantly, HOM/TG mice have circulating liver enzyme levels and liver sizes comparable to WT mice, and no induction of p53 or p21 (Fig. 6a-c). Polysome analysis also showed that the *BRF1* transgene rescued the translational defects seen with *Brf1* deletion (S Fig. 6).

**Fig. 7:**
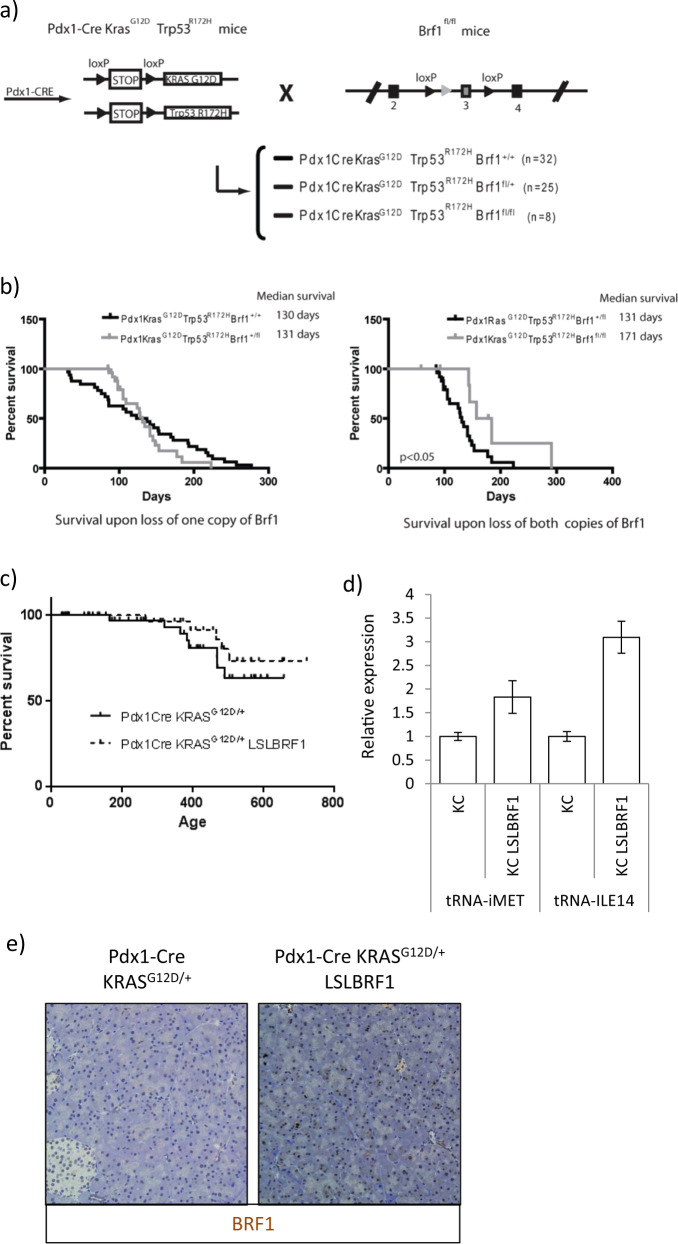
Brf1 is essential during pancreas development. **a** Schematic showing the crosses performed to obtain the respective cohorts to assess the effects of *Brf1* deletion in a mouse model of PDAC. **b** Kaplan–Meier curves showing pancreatic tumor-free survival in *KPC Brf1*^*+/+*^ (*n* = 32) vs *KPC Brf1fl/* *+* (*n* = 26) mice (left panel) and for *KPC Brf1*^*fl/+*^ (*n* = 26) vs *KPC Brf1*^*fl/fl*^ (*n* = 8) mice (right panel). Median survival for each genotype is indicated. **c** Kaplan–Meier survival curves showing pancreatic tumor-free survival in KC (*Pdx1-Cre Kras*^*G12D/+*^, continuous line, *n* = 44) vs KC HPRT^LSL-BRF1^ mice (*Pdx1-Cre Kras*^*G12D/+*^ HPRT^LSL-BRF1^, disrupted line, *n* = 33). **d** tRNA^iMET^ and tRNA^ILE14^ expression in pancreata from KC (*Pdx1-Cre Kras*^*G12D/+*^) and KC LSLBRF1 mice (*Pdx1-Cre Kras*^*G12D/+*^ HPRT^LSL-BRF1^) mice at 6 weeks post induction. *N* = 3 per genotype. **e** Staining for BRF1 in pancreata from KC (*Pdx1-Cre Kras*^*G12D/+*^) and KC HPRT^LSLBRF1^ mice (*Pdx1-Cre Kras*^*G12D/+*^ HPRT^LSL-BRF1^) mice at 6 weeks post induction

Next, we analysed whether acute overexpression of *BRF1* induces proliferation. No robust phenotype was observed in terms of proliferation or apoptosis in the liver of *AhCre Hprt*^*LSL-BRF1*^ mice at 8dpi. (S Fig. 7A-C). Moreover, when *Hprt*^*LSL-BRF1*^ mice were crossed to animals expressing ubiquitous *CAAG-Cre*^*ER*^ to activate BRF1 across the mouse, no gross phenotypes were observed (data not shown).

### BRF1 heterozygosity does not alter tumorigenesis

Next we analysed the impact of *Brf1* upon intestinal tumorigenesis. Previous work has shown that c-Myc stimulates *Brf1* and Pol-III-mediated transcription. *C-Myc* is a target of the WNT signalling pathway, and haploinsufficiency for *c-Myc* can slow *Apc* loss-mediated intestinal tumorigenesis [51]. We therefore crossed *Brf1*^*fl/+*^ mice to *AhCre Apc*^*fl/+*^ mice, which develop tumours upon loss of the remaining *Apc* allele.

Cohorts of *AhCre Apc*^*fl/+*^ *Brf1*^*+/+*^ and *AhCre Apc*^*fl/+*^ *Brf1*^*fl/+*^ were aged until they developed signs of intestinal neoplasia. Heterozygous loss of *Brf1* did not alter tumor development (S FIG 8A) or tumor number or size (S FIG 8B), suggesting that Brf1 is not limiting for intestinal tumorigenesis. Importantly, following heterozygous deletion of *Brf1* in the liver for 10 days we saw a 50% reduction in *Brf1* mRNA, but no difference in Brf1 protein or expression Pol-III targets (S Fig. 4). It was not possible to analyse Brf1 protein expression within intestine due to its multicellular nature; *AhCre* driven recombination only deletes genes in a fraction of the tissue. Nevertheless, we conclude that heterozygous deletion of Brf1 in the intestine does not limit tumorigenesis.

We next analysed the role of Brf1 in pancreatic tumour development where heterozygosity for *c-Myc* strongly suppresses pancreatic cancer formation [52]. We crossed *Brf1*^*fl/+*^ mice to the KPC mouse model (*LSL-Kras*^*G12D*^, *LSL-Trp53*^*R172H*^ with *Pdx1-Cre*). These KPC mice develop pancreatic ductal adenocarcinoima with a median incidence of 4 months [53]. Activation of KRAS and loss of p53 increases Pol-III activity in other tumor settings [24, 54].

We generated and aged cohorts of KPC mice either wild-type, heterozygous or homozygous for deletion of *Brf1* (Fig. 7a). There was no change in survival between KPC and KPC *Brf1*^*fl/+*^ mice; however, there was a significant delay in PDAC formation in KPC *Brf1*^*fl/fl*^ mice (Fig. 7b) where survival was extended by 40 days compared to KPC mice. The pancreatic tumours that developed in KPC *Brf1*^*fl/fl*^ mice were similar in morphology to those of KPC and KPC *Brf1*^*fl/+*^ mice.

*Pdx1-Cre* expression is mosaic in the developing pancreas. Thus, it is possible that non-recombined cells preferentially populate the pancreas, as previously observed [55]. We found that tumours from KPC *Brf1*^*fl/fl*^ mice showed a lack of recombination at the *Brf1* locus (S FIG 10A). Therefore the difference in survival is not due to any effect of Brf1 on tumorigenesis but rather the time taken for establishment of Brf1-proficient pancreata.

Consistent with this, *Pdx1-Cre Brf1*^*fl/fl*^ were lived for over 300 days (data not shown). Given the embryonic lethality upon loss of *Brf1* and the apparently normal pancreata of *Pdx1-Cre Brf1*^*fl/fl*^ mice, we examined if these were also composed of non-recombined cells. *Pdx1-Cre Brf1*^*fl/fl*^ mice carrying the *Rosa26*^*LSL-RFP*^ reporter allele did not show recombination (S FIG 10B) with no recombination confirmed by PCR for the *Brf1* locus (S FIG 9). Thus, deletion of *Brf1* in the pancreas is not conducive with its development.

We therefore examined the impact of BRF1 overexpression in a longer latency (350 days) pancreatic model: *LSL-Kras*^*G12D/+*^ *Pdx1-Cre* (KC), crossing this with the human *Hprt*^*LSL-BRF1*^. We saw no difference in survival between KC and KC *Hprt*^*LSL-BRF1*^ (Fig. 7c). Overexpression of BRF1 was confirmed and increased tRNA^iMET^ and tRNA^ILE14^ expression at 6 weeks post induction (Fig. 7d, e). Together this suggests that Brf1-dependent Pol-III activity is not limiting for pancreatic tumorigenesis.

Interestingly, overexpression of BRF1 in wild-type livers did not restore tRNA expression (S Fig. 5) in Brf1^fl/fl^ mice but did increase tRNA expression in Kras mutant pancreas (Fig. 7d). This may be due to tissue specific differences in Pol-III regulation or a role for mutant Kras in tRNA transcription. Mutant Ras promotes tRNA synthesis via Brf1 in *Drosophila* [56], providing a possible explanation for the enhanced tRNA transcription in BRF1 over-expressing, Kras mutant pancreata.

## Discussion

Pol-III activity is upregulated in cancer and is often associated with oncogenic driver mutations [16, 22, 38]. Despite this, few studies have addressed whether increased Pol-III activity overcomes a functional limitation for Pol-III. It is also unclear whether there is a therapeutic window for targeting Pol-III. Therefore, we engineered mice to specifically up or downregulate Brf1, in both normal and cancerous cells, to address the role of Pol-III in vivo.

We show that BRF1 is essential for organ homeostasis, but not limiting in selected instances of tumorigenesis. Constitutive knockout of *Brf1* leads to a pre-implantation defect due to an impaired passage from morulae to blastocyst at 3.5dpc. There is an increase in energy demand during at this point of embryo development, and the embryo is large enough to dilute maternal mRNAs [36]. Pol-III activity has been detected as early as the 2 cell embryo [57]. Increased growth and energy demand during the passage from morulae to blastocyst, coupled with a possible dilution of maternal *Brf1* message, makes *Brf1* essential at 3.5dpc. Development was the only instance of a phenotype from *Brf1* heterozygosity. However, as *Brf1* heterozygous mice were viable and of normal size there may be compensatory mechanisms during development.

Within 3 days of deleting *Brf1* in the crypts of the small intestine we observed an increase in p53, p21, γH2AX protein and apoptosis. Therefore, in a rapidly dividing epithelium there is an immediate requirement for Pol-III activity, and if not present, a p53 response is activated, likely due to the preceding reduction in the protein synthesis. It is worth noting that even in times of translational stress, specific translation can be induced [58]. The gut is especially sensitised to p53-induced death, as has been observed following deletion of CHK1 or BRCA2 [59, 60]. We observed rapid repopulation of the intestine with non-recombined cells, which precluded characterisation of the impact upon translation. This is a well-known phenotype of the intestinal epithelium and is aided by the process of neutral drift, where a single crypt stem cell can repopulate the entire crypt [61, 62].

The liver provided a more tractable system to investigate the effects of *Brf1* loss. Liver epithelial turnover is slower and effective recombination is possible in a potentially regenerative epithelium [63, 64]. In *Brf1* knockout livers there was an induction of p53 at day 4, becoming more prominent at day 6 and day 8, when p21 and γH2AX were subsequently induced. p53 induction followed the reduction in polysome associated mRNAs, with a mild reduction at day 4, which progressed to an almost complete absence of polysomes by day 8. This ultimately led to liver failure as evidenced by jaundice, liver architectural disturbance and clinical deterioration. Given the kinetics of p53 induction and the polysome profiles, we suggest that reducing Pol-III activity decreases protein synthesis, which in turn acts as a “checkpoint” to signal the upregulation of p53. It is also noted that while the overall rate of protein synthesis falls after *Brf1* deletion we again see a marked induction of p53 and p21. This phenomenon suggests preferential translation of certain transcripts or a reduced degradation of certain proteins.

Finally, we show that Brf1 is also essential during pancreatic development using *Pdx1-Cre*. *Pdx1* is expressed from 8.5dpc and plays a role in differentiation of all pancreatic lineages. Adult pancreata from *Pdx1-Cre Brf1*^*fl/fl*^ appeared normal but were composed exclusively of non-recombined cells, arguing for a strong negative selection against Brf1 deletion.

We hypothesised that *Brf1* heterozygous mice would show suppression of tumour initiation and development. However, outside a possible impact during embryogenesis, we found no effect in homeostasis for three epithelia (pancreas, intestine and liver), or tumorigenesis for two epithelia (pancreas and intestine). Given difficulties in detecting mouse Brf1 protein levels it is possible that heterozygotes might maintain protein levels similar to wild-type. Indeed, we have shown this to be the case following heterozygous deletion in the liver. Conversely, overexpression of BRF1 did not initiate or promote tumorigenesis. Therefore, we were unable to find a limiting role for Brf1 activity in cancer despite the large number of studies showing Pol-III deregulation. Most of the work investigating Pol-III activity has compared normal cells to late stage cancer cells. Therefore there has not been a stage-specific characterisation of the timing of BRF1 and Pol-III activity upregulation during carcinogenesis. Thus, it may be that oncogenes such as c-*MYC*, activate Pol-III expression during tumor initiation, mimicking developmental and homeostatic scenarios where Pol-III activity is required. Consistent with this a recent study showed a progressive increase in Pol-III activity in a model of breast cancer initiation and progression, which correlated with c-MYC activity [65]. However, levels of Pol-III activity are not limiting at these stages and increasing Pol-III activity per se in the absence of an oncogenic event is insufficient to drive proliferation and growth. This differs to the *Drosophila* scenario, where BRF1 is required for normal pupa development [29, 30], and may reflect increased mechanisms to control growth in longer lived mammals. Indeed, the oncogenic potential of BRF1 has recently been questioned with loss-of-function mutations in BRF1 potentially actually responsible for some heritable colorectal cancers [66]. Similarly, high BRF1 expression is a favourable prognostic marker in breast cancer [67].

Finally, it is possible that increased Pol-III activity in cancer may be important beyond proliferation and growth. Most work comparing normal and cancer cells involves late stage aggressive tumor cells. Thus, Pol-III activity may be important in invasion, migration and angiogenesis. Indeed, two recent reports show that Pol-III could be involved in tumour cell migration and metastasis [44, 45]. It is interesting to note that small molecules targeting Pol-I can specifically target cancer cells while sparing normal counterparts [68]. Approaches to reduce BRF1 beyond heterozygosity might be required to reveal a limiting function and be tolerated by normal cells.

In summary, we have revealed a vital role for BRF1 in development and homeostasis. However, we found little evidence for a role for Brf1 as a driver of cancer as tumor and normal cells appear equally reliant on its fundamental activity. Moreover, Pol-III activity alone is not sufficient to transform cells or drive proliferative or growth phenotypes in the epithelia studied.

## Materials and methods

### Genetically modified mice and animal care

Animals were kept in conventional animal facilities and experiments were carried out in compliance with U.K. Home Office guidelines (ASPA 1986 & EU Directive 2010). Mice were genotyped by Transnetyx INC. (Cordova, Tennessee). *Brf1*^fl/+^ heterozygous mice were crossed to Deleter-Cre recombinase [46] to excise exon 3 and generate the knockout allele. The *Brf1*^fl/+^ and HPRT^LSL-BRF1^ mice were also crossed with the Pdx1-Cre and Ah-Cre mouse strains previously described [48, 69]. To induce recombination in the Ah-Cre mice three intraperitoneal injections of β-naphthoflavone (80 mg/kg) were given at 4 h intervals. For AAV induction, mice were injected with virus particles 2 × 10^11^ genetic copies (GC)/mouse of either AAV8.TBG.Cre.Rbg (UPenn Vector Core, #: AV-8-PV1091) vector or AAV8.TBG.PI.null.bGH (control virus) (UPenn Vector Core, #: AV-8-PV0148) via tail vein in 100 µl PBS as previously described [70].

### Generating Brf1 flox mice

Mice carrying the *Brf1* flox allele were generated by Taconic Artemis (Cologne, Germany) according to their standard procedures.

### Generating Brf1 TG mice

Conditional BRF1 expressing mice were generated by targeting a human *BRF1* cDNA under the Cre-dependent control of a CAAG promoter to the expression-permissive HPRT locus [71]. The targeting vector was generated essentially as described by [72] but with a cDNA encoding the full length human BRF1 protein cloned downstream of the lox-stop-lox. The vector was linearised and electroporated into Hprt-deficient HM1 ES cells, cultured on a DR4 mouse embryonic fibroblast feeder layer [73, 74].

Homologous recombinants were selected in medium containing HAT supplement (Sigma). Correct targeting of the vector to the Hprt locus on both the 5′, and 3′ sides was confirmed using PCR on genomic DNA. Genotyping was performed by PCR using Expand Long Template (Roche) according to the manufacturer’s recommendations. Primers used for genotyping targeted ES cells were 5′: GTTGCTGAGGCAAAAATAGTGTAAT and CCATTTACCGTAAGTTATGTAACGC and 3′: CTACCTAGTGAGCCTGCAAACTG and ATGTAAGTGCTAGGAATTGAACCTG.

Following identification of correctly targeted clones, derived by injection of targeted mESC into C57BL/6 J blastocysts according to standard protocols [75]. Germline transmission was identified by coat colour and transmission of the transgene confirmed by PCR.

### In situ hybridisation

Single strand probes labelled with digoxigenin were generated from a linearised tRNA^iMET^ gene using DIG RNA labelling kit (Roche) according to the manufacturer’s specifications. RNA dot blot analysis was used to normalise the sense and antisense riboprobes. Hybridization was carried out in a sealed, humidified container at 65 °C for 24 h with 0.5 ug/ml riboprobe and 50 ug/ml yeast tRNA in 50% deionised formamide, 5X SSC (pH 4.5), 2% blocking powder (Roche), 0.5% CHAPS, 1x Denhardt’s solution (Sigma), 10% Dextran sulphate, 1 μg/μL salmon sperm DNA (Sigma) and 5 mM EDTA. After hybridization, sections were rinsed once in 2X SSC (pH 4.5) followed by 3 washes of 20 min at 65 °C in 2X SSC/50% formamide. Sections were then rinsed 5 times in TBS and blocked for 1 h in 0.5% blocking powder (Roche) in TBS. Signal was detected using DIG Nucleic Acid Detection Kit (Roche) according to the manufacturer’s specifications. BaseScope analysis was carried out according to the manufacturer’s instructions (ACD Bio) using a probe designed to target bases 548-633 of mouse *Brf1*.

### Immunohistochemistry

Tissues were fixed in 10% neutral buffered formalin for no longer than 24 h before being processed into paraffin blocks according to standard protocols. For harvesting of the intestine samples, small intestines removed and flushed with water. The first 5 cm of intestine were divided into 1-cm lengths, bundled using surgical tape, and then fixed in 4% formaldehyde at 4 °C for no more than 24 h before processing. Tissue sections (5 µm) were either stained using haematoxylin and eosin (H&E), for histological analysis, or were used for immunohistochemistry (IHC) using standard methods. Antibodies used for IHC are in Supplemental Table 2 together with the appropriate dilutions for use. For each antibody, staining was performed on at least three mice of each genotype. Representative images are shown for each staining.

### Crypt size and apoptosis assay

Apoptosis and crypt size were scored from H&E stained sections as previously described [76]. For each analysis, 25 full crypts or 50 half crypts were scored from at least three mice of each genotype.

### Harvesting and genotyping 2.5dpc and 3.5dpc embryos

Embryos at 8-cell and blastocyst stage were collected by dissecting and flushing oviducts with M2 medium (M7167, Sigma–Aldrich) as described [75]. At 3.5dpc embryos were genotyped via conventional PCR using a combination of oligo1, oligo 2 and oligo 3 primers, sequences shown in Supplemental Table 2. Embryos were transferred directly in the PCR mixture and loaded into Bio-Rad DNA Engine DYAD Thermal Cycler with the following cycling parameters 95 °C 10 min, 30 cycles of 95 °C 30 s, 60 °C 30 s, 72 °C 30 s and 72 °C 10 min. PCR products were separated and visualised on an agarose gel.

In order to carry out the in vitro growth assay each of the 3.5dpc embryos was grown in a separate well of a gelatine-coated 96-well plate in DMEM media supplemented with 15% Fetal Calf Serum and non-essential amino acids. Pictures of each embryo were taken each day for 5 days using an Olympus CKX41 light microscope with a Qimageing Fast 1394 camera and QCapture Pro visualisation program. After 5 days the embryos were trypsinised off the 96-well plate and genotyped via conventional PCR described above. 2.5dpc embryos were pooled together and grown in vitro using M2 medium for 2 days. At the end of the two days each embryo was separated and genotyped using conventional PCR.

### Immunoblotting

Western Blot analysis was performed as described previously with antibody A301-228A (Bethyl) against BRF1 [17].

### PCR and gel electrophoresis

PCR was conducted using TAQ polymerase from Invitrogen (1034020) as per manufacturer’s instructions. DNA for reactions was isolated from pancreatic tumours using the QiaAmp DNA Blood Mini Kit (Qiagen) as per manufacturer’s instructions. 0.1 ng of DNA was used in each reaction together with a combination of 0.5 mM oligo1 and either oligo 2 or oligo 3 primers, as per figure legend, and 0.5 mM β-actin primers as control. A Bio-Rad DNA Engine DYAD Thermal Cycler was used with the following cycling parameters 95 °C 10 min, 25 cycles of 95 °C 30 s, 60 °C 30 s, 72 °C 30 s and 72 °C 10 min. Samples were loaded in a polyacrylamide gel and visualised with Sybr Safe as per manufacturer’s instructions using a Gene Genius Bioimaging system with a Syngene GeneSnap visualisation program.

### Quantitative real-time PCR

Quantitative real time PCR was performed using PerfeCTa SYBR Green FastMix (Quanta BioSciences) according to the manufacturer’s protocol. Analysis was performed on the C1000 Thermocycler CFX96 Real Time System (Bio-Rad). Relative quantification was determined from a standard curve using the Bio-Rad CFXManager software (Version 1.5.534.011). Primers used are described in Supplemental Table 1. For each reaction 0.5 mM of forward and reverse primer mixture was used. Three step PCR cycling parameters were used in all reactions as follows 95 °C for 15 min, 35 cycles of 95 °C for 10 s, 60 °C 10 s and 72 °C for 10 s followed by a melting curve.

### Polysome profiling

To generate tissue for polysomal profile analysis, the whole liver of the mouse was perfused with 0.1 mg/ml cycloheximide (Sigma) in PBS and incubated with 0.1 mg/ml cycloheximide in HBSS (Gibco) with 10 mM EDTA for 5 min at 37 °C. Livers were cut into small pieces and used for downstream analysis.

Liver pieces were lysed in ice cold 300 mM NaCl, 15 mM MgCl_2_, 15 mM Tris (pH 7.5) containing 500 units/ml RNAsin, 1 mg/ml heparin sulphate and 0.1 mg/ml cycloheximide supplemented with 0.1% (v/v) Triton X-100. Post-nuclear lysates were layered on ∼10 ml 10–50% (w/v) sucrose gradients of the same buffer omitting Triton X-100. Gradients were centrifuged at 38,000 rpm for 3 h at 4 °C in a SW40Ti rotor (Beckman Coulter) and separated through a live OD254nm UV spectrometer (Isco).

### Blood analysis

Blood was collected via heart puncture immediately upon euthanizing the animal. Blood samples were processed for a full liver profile using an Olympus Au640 (Beckman Coulter) as per manufacturer instructions.

### Supplementary information

Supplemental figures

Supplemental tables

Supplemental Figure legends
