# Supplementary material for: Brf1 loss and not overexpression disrupts tissues homeostasis in the intestine, liver and pancreas
Source: Cell Death Differ. 2019 Mar 11;26(12):2535–50. doi: 10.1038/s41418-019-0316-7 (PMC6861133; doi:10.1038/s41418-019-0316-7)
Supplement: Supplementary file 1 — Supplemental figures [file 41418_2019_316_MOESM1_ESM.pdf]

S Fig 1: Confirming the generation of a *Brf1* floxed allele

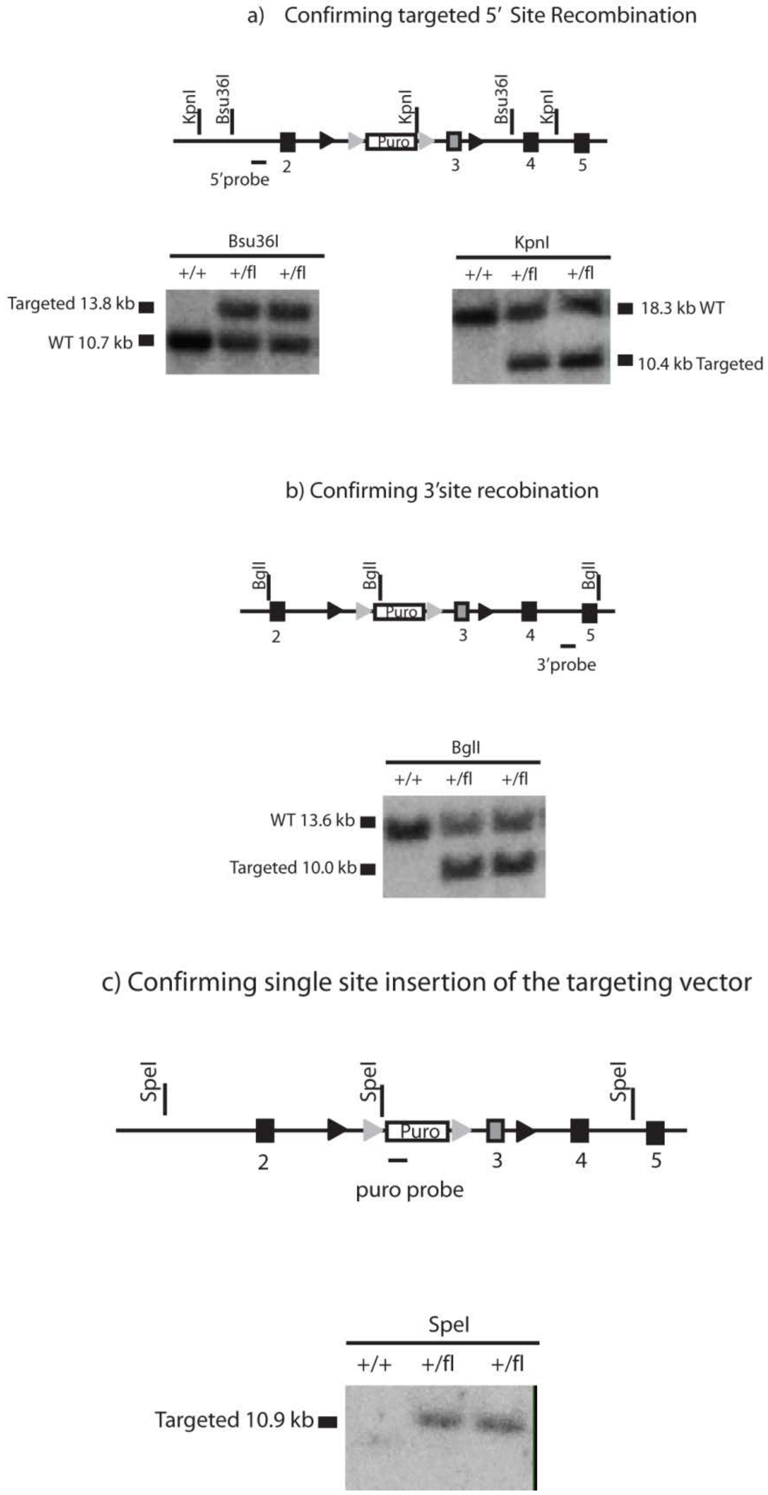

S Fig 2: Generating and assessing *Brf1* heterozygous mice

a): Generating Brf1 heterozygous mice

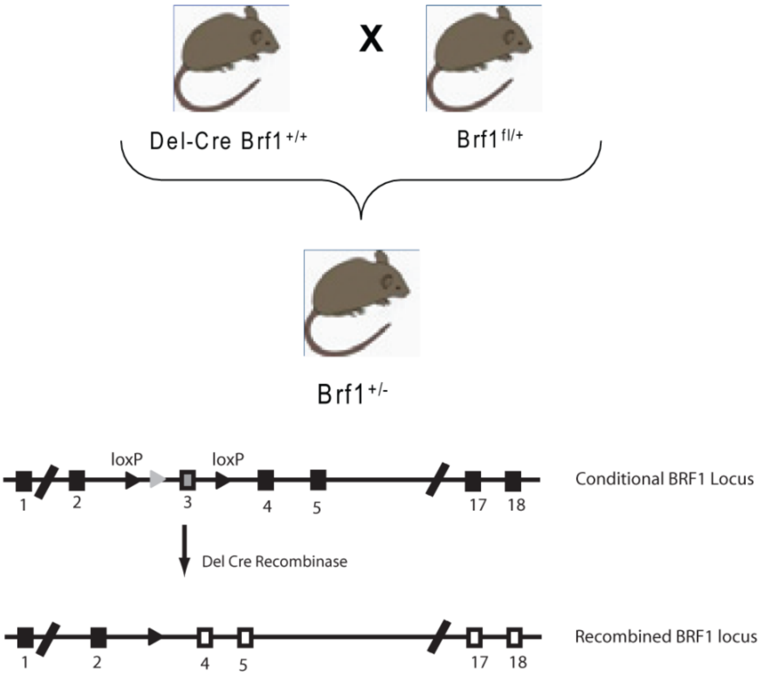

b) *Brf1* +/- display no change in overall animal weight

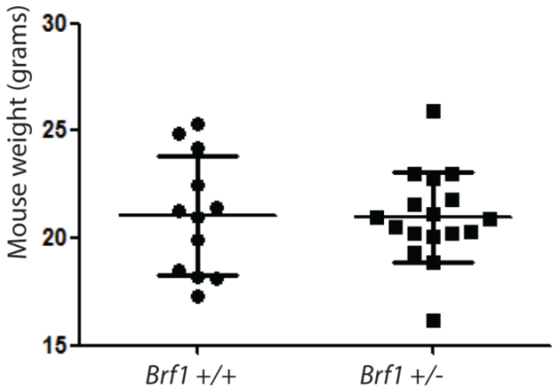

S Fig 3: Levels of γH2AX, cleaved caspase 3 and pan-cytokeratin upon loss of *Brf1*

Liko_SFig3

a) Deletion of Brf1 induces γH2AX in the liver after 6 days of induction

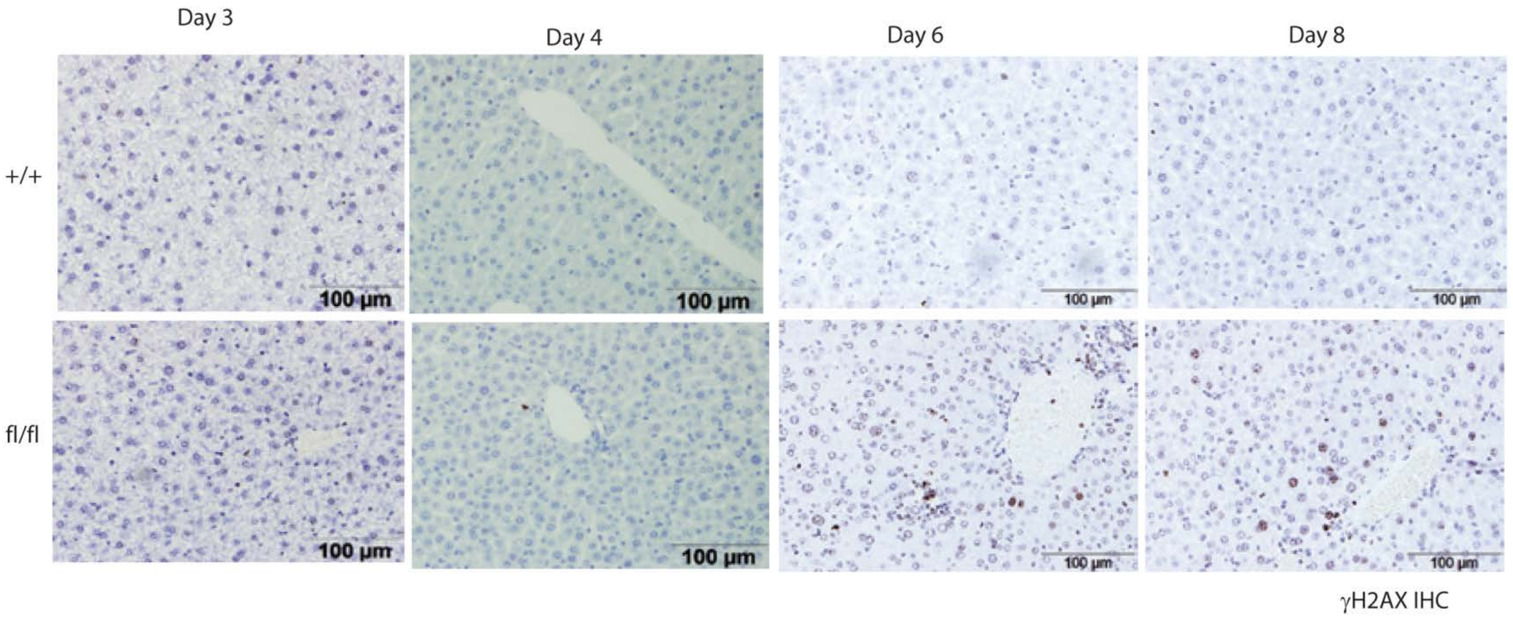

b) Deletion of Brf1 promotes cleaved caspase 3 and cytokeratin induction in liver

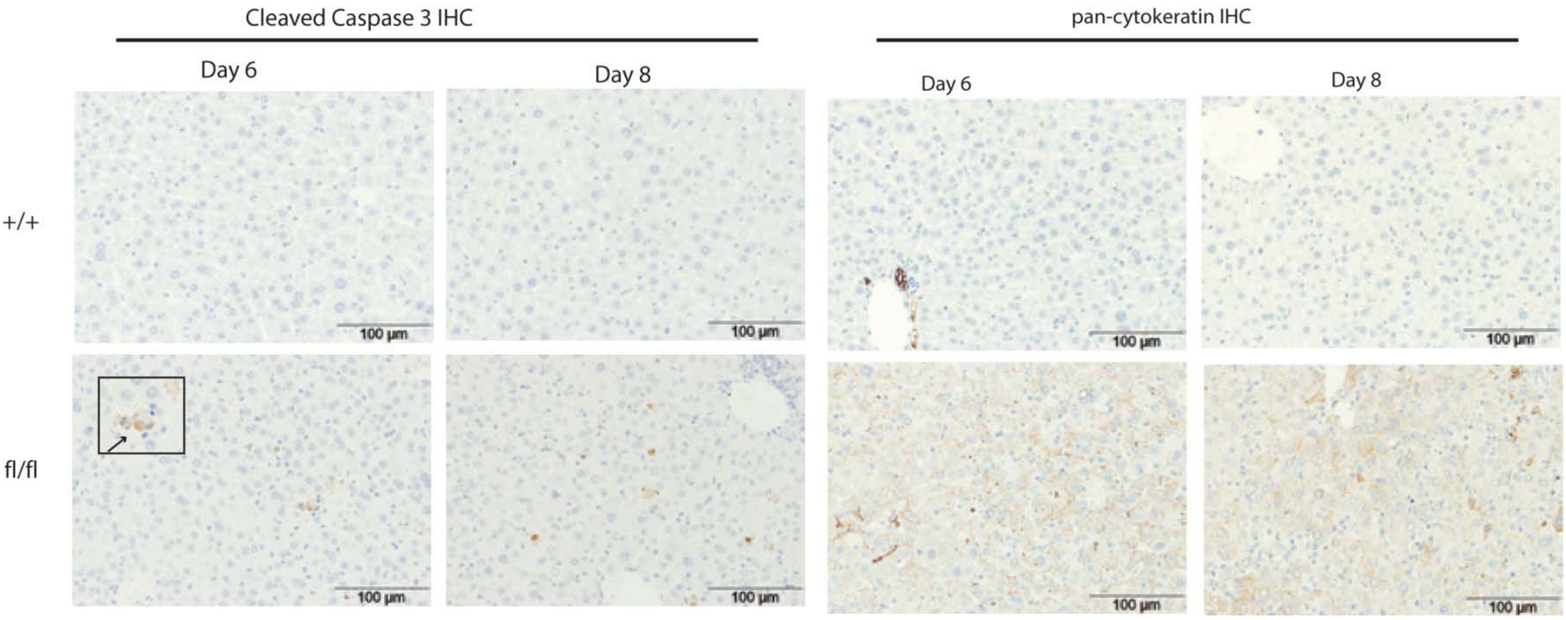

S Fig 4: Acute deletion of Brf1 in hepatocytes causes a liver collapse phenotype

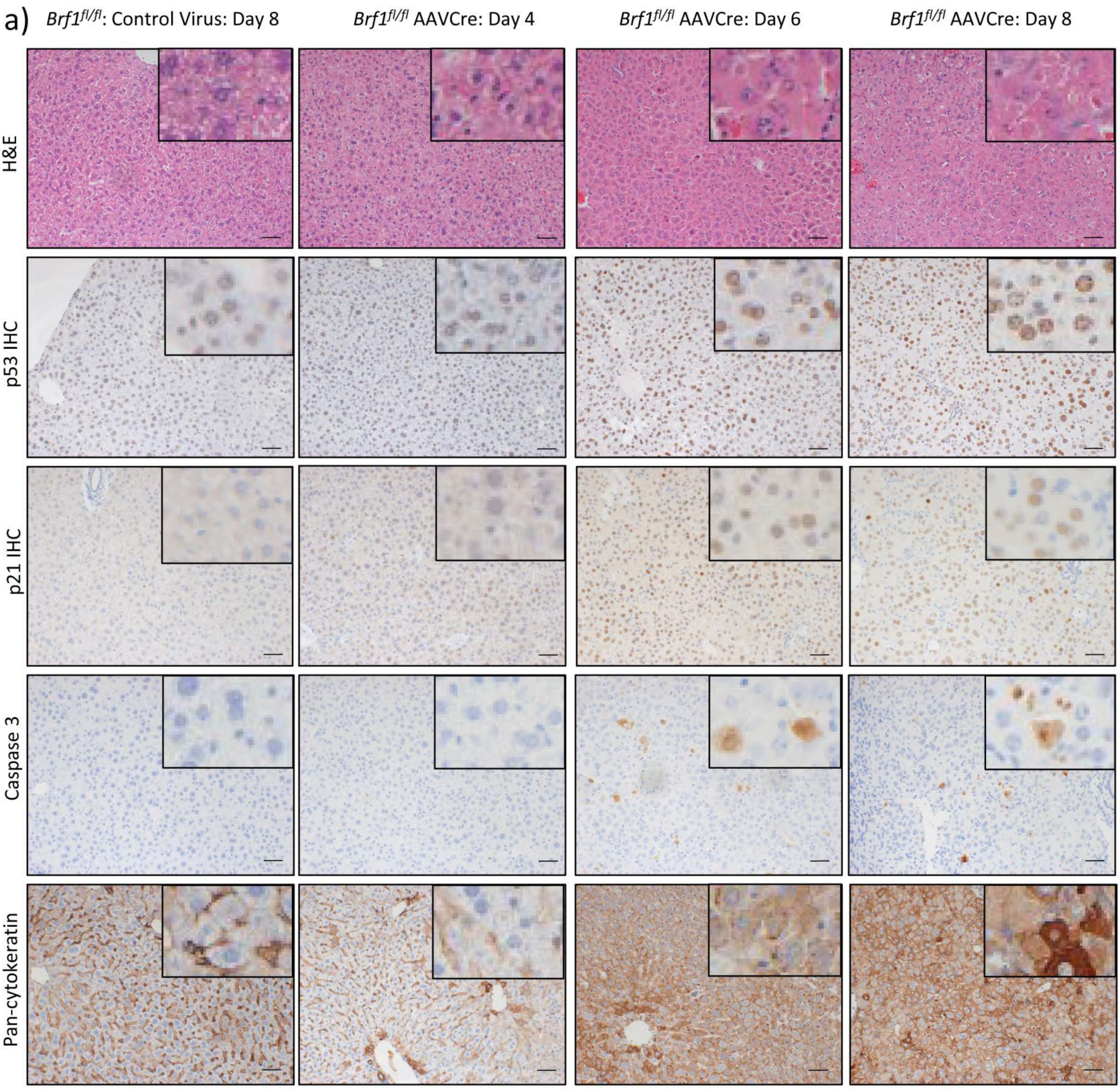

b)

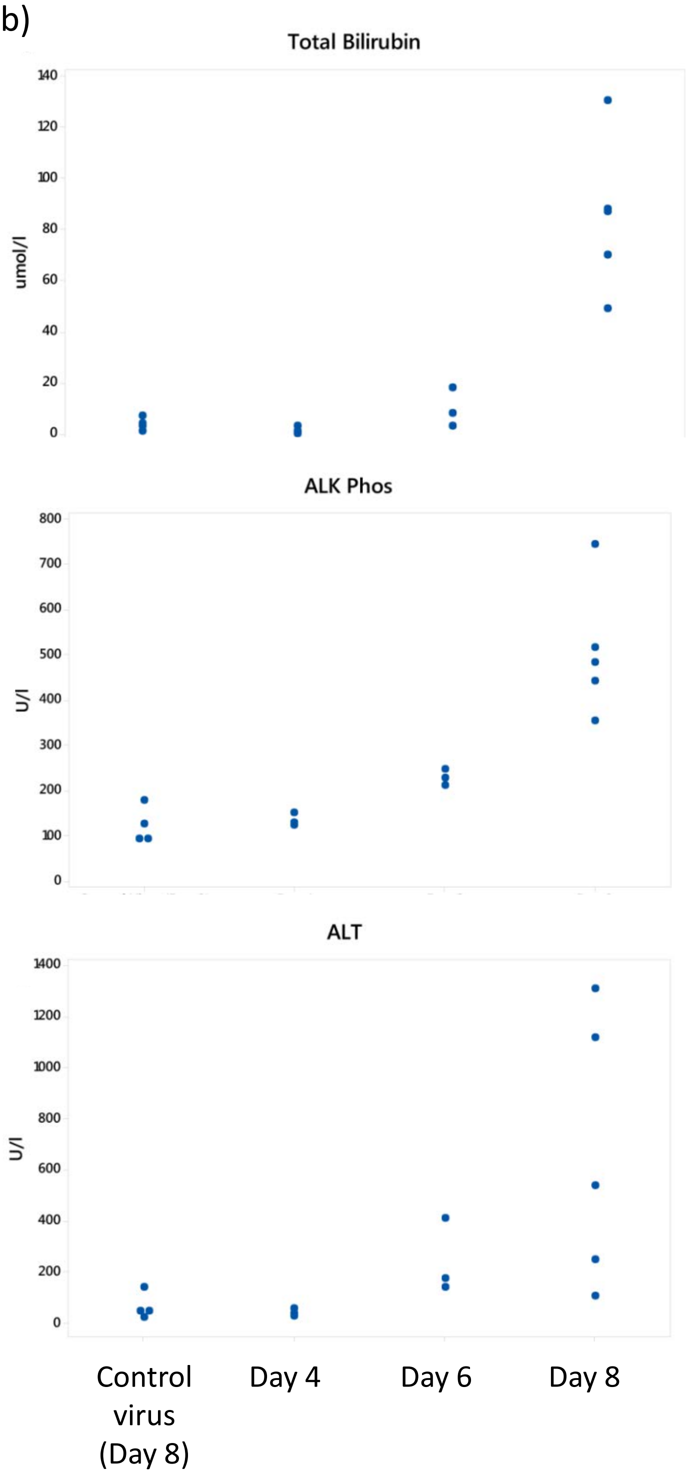
Total Bilirubin
umol/l
140
120
100
80
60
40
20
0
ALK Phos
U/l
800
700
600
500
400
300
200
100
0
ALT
U/l
1400
1200
1000
800
600
400
200
0
Control virus (Day 8)
Day 4
Day 6
Day 8

c)

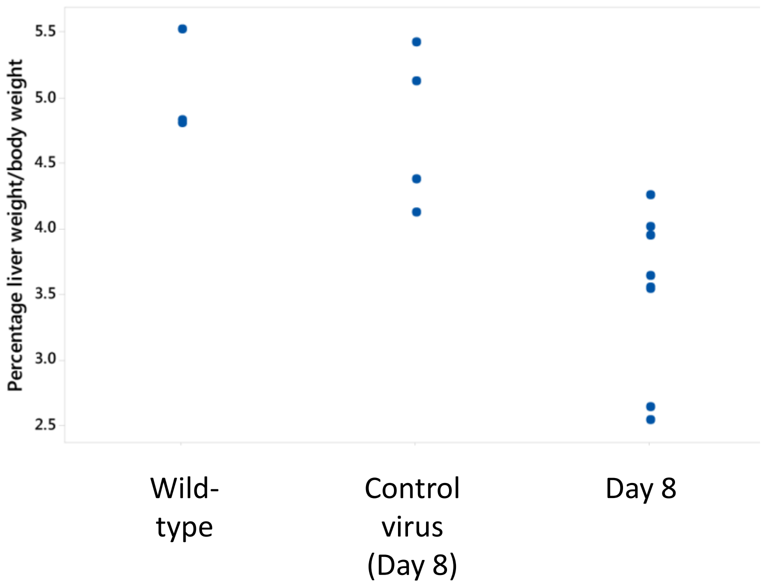
Percentage liver weight/body weight
5.5
5.0
4.5
4.0
3.5
3.0
2.5
Wild-type
Control virus (Day 8)
Day 8

d)

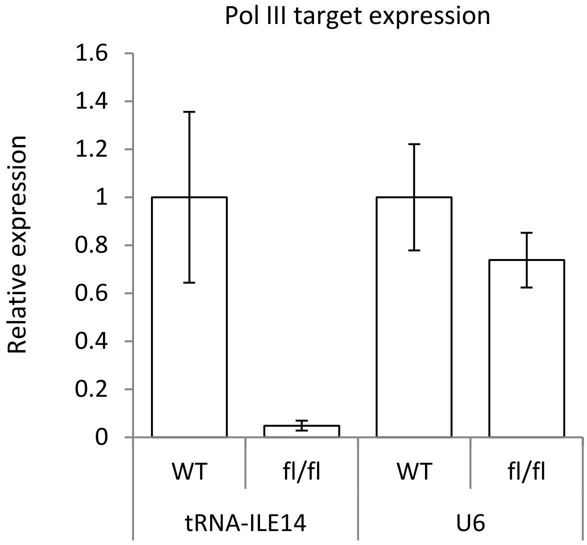
Pol III target expression
Relative expression
1.6
1.4
1.2
1
0.8
0.6
0.4
0.2
0
WT
fl/fl
WT
fl/fl
tRNA-ILE14
U6

e)

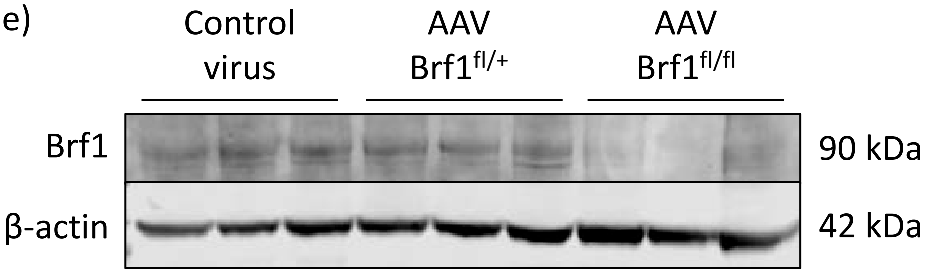
Control virus
AAV Brf1fl/+
AAV Brf1fl/fl
Brf1
β-actin
90 kDa
42 kDa

a) Brf1 mRNA expression is increased by the human transgene

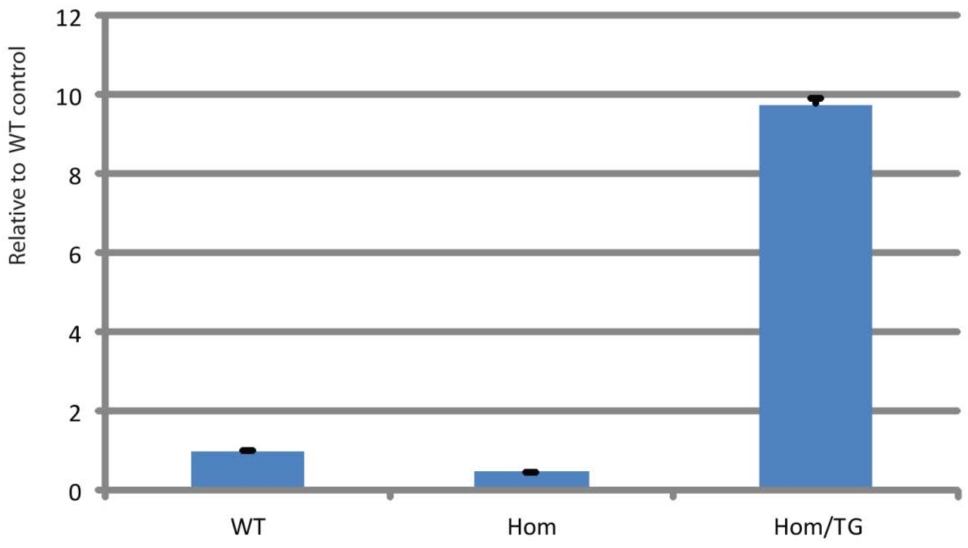

b) Human BRF1 rescues the loss of tRNAs upon *Brf1* deletion

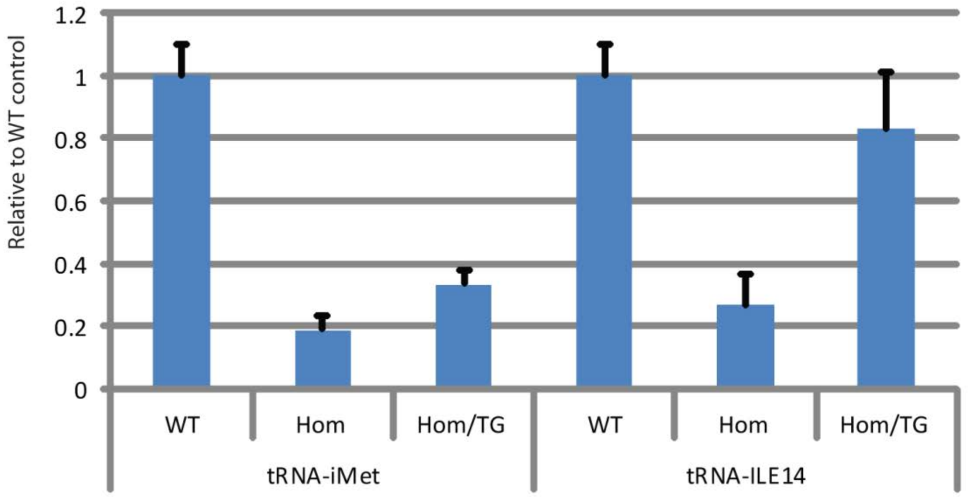

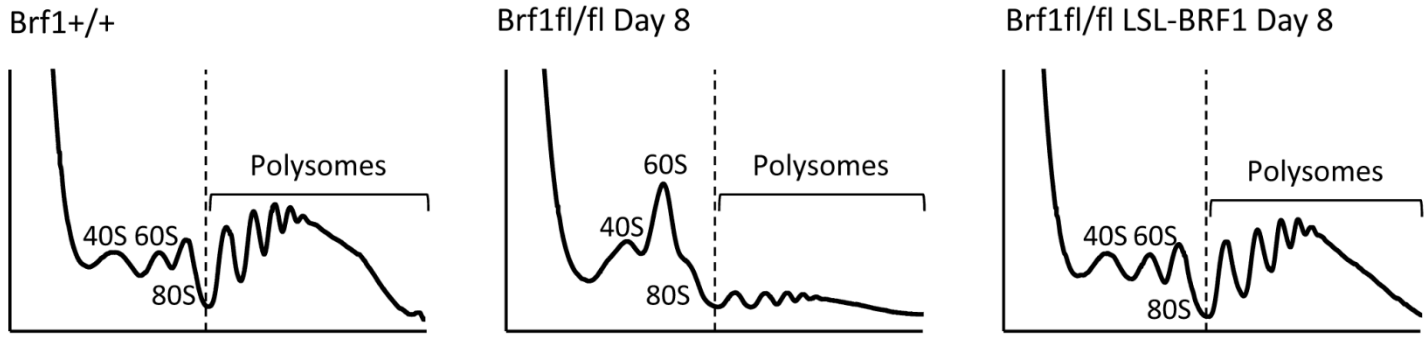
Brf1+/+
40S 60S
80S
Polysomes
Brf1fl/fl Day 8
60S
40S
80S
Polysomes
Brf1fl/fl LSL-BRF1 Day 8
40S 60S
80S
Polysomes

S Fig 7: Human BRF1 expression does not induce proliferation of hepatocytes

a) Morphology of Brf1 overexpressing livers 8 days after induction

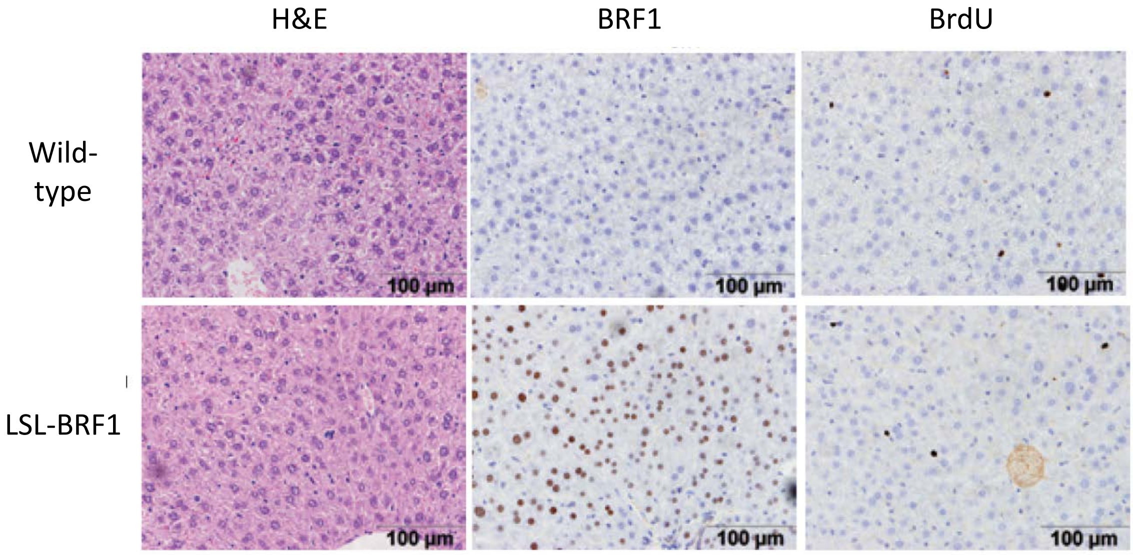

b) Quantification of BrdU positive cells in the liver

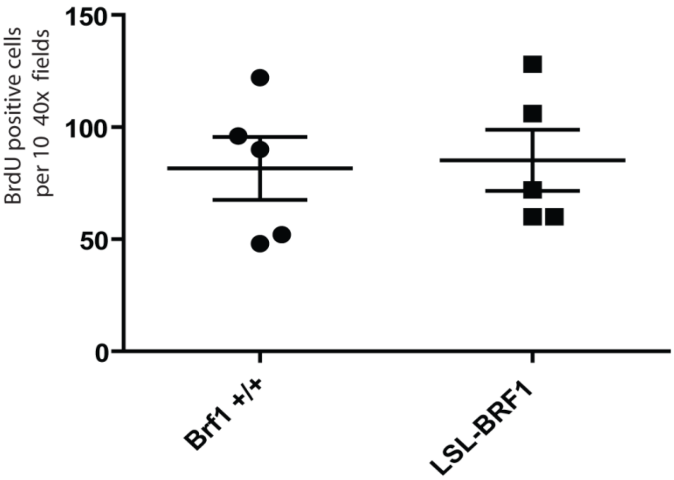

c) Liver weights of BRF1 overexpressing mice

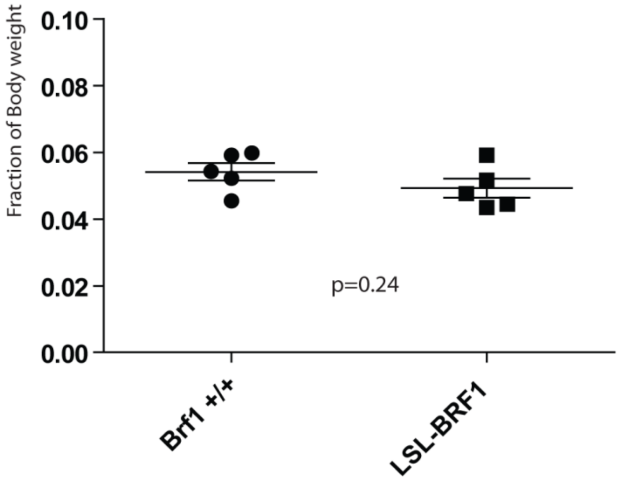

S Fig 8: *Brf1* heterozygosity does not affect an intestinal tumorigenesis model

a) Survival from intestinal tumours of APC Brf1+/+ and APC Brf1 +/- mice is unchanged

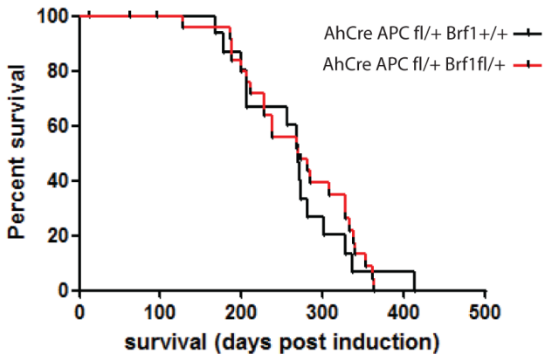

b) Losing one copy of Brf1 does not affect average tumour number and size in APC mice

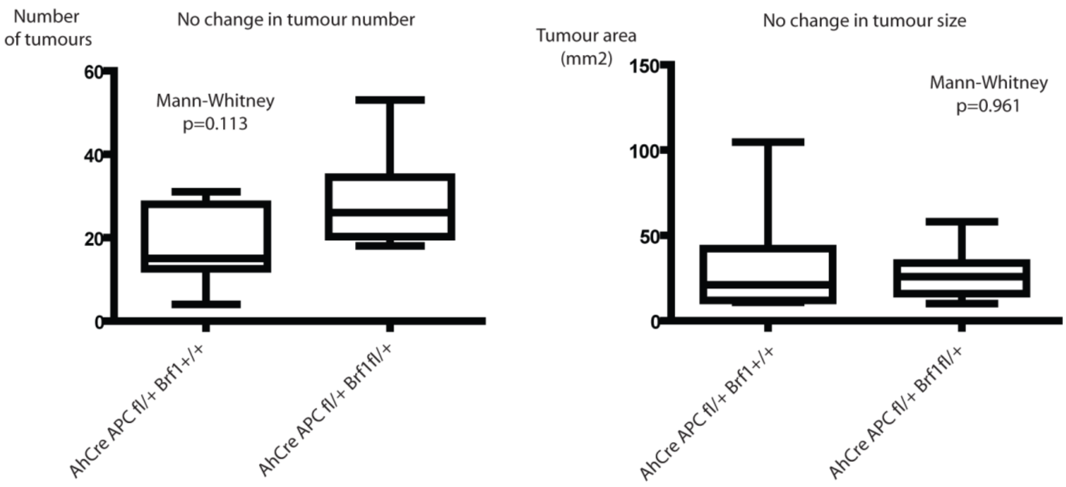

c)

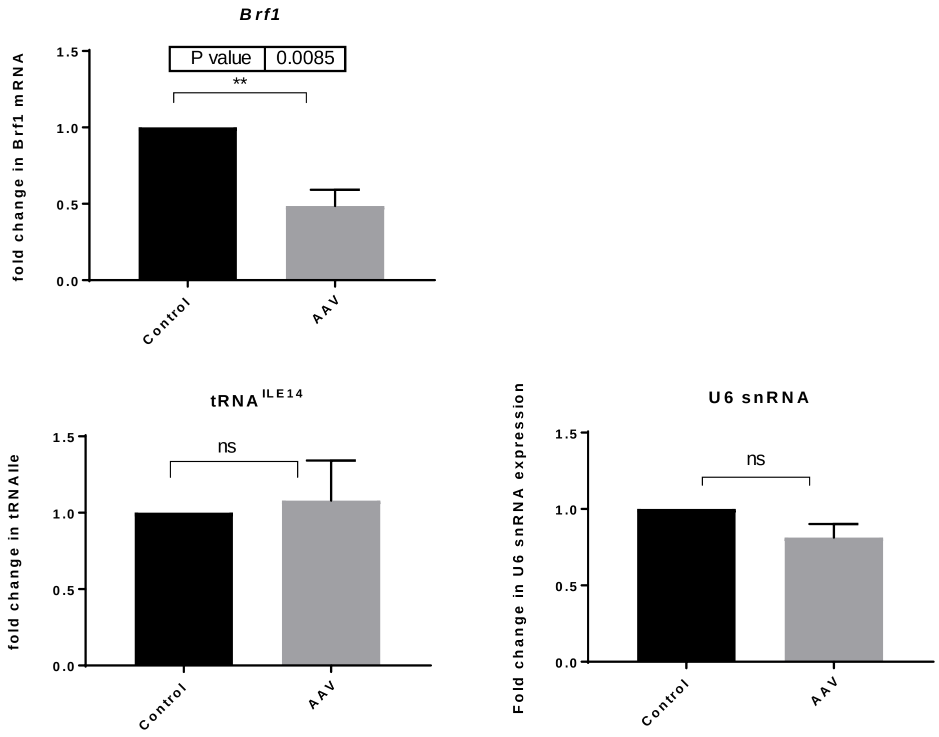

S Fig 9: Deletion of Brf1 is detrimental to the developing pancreas

Pancreas of Pdx1-Cre Brf1$^{fl/fl}$ mice arises from unrecombined precursors

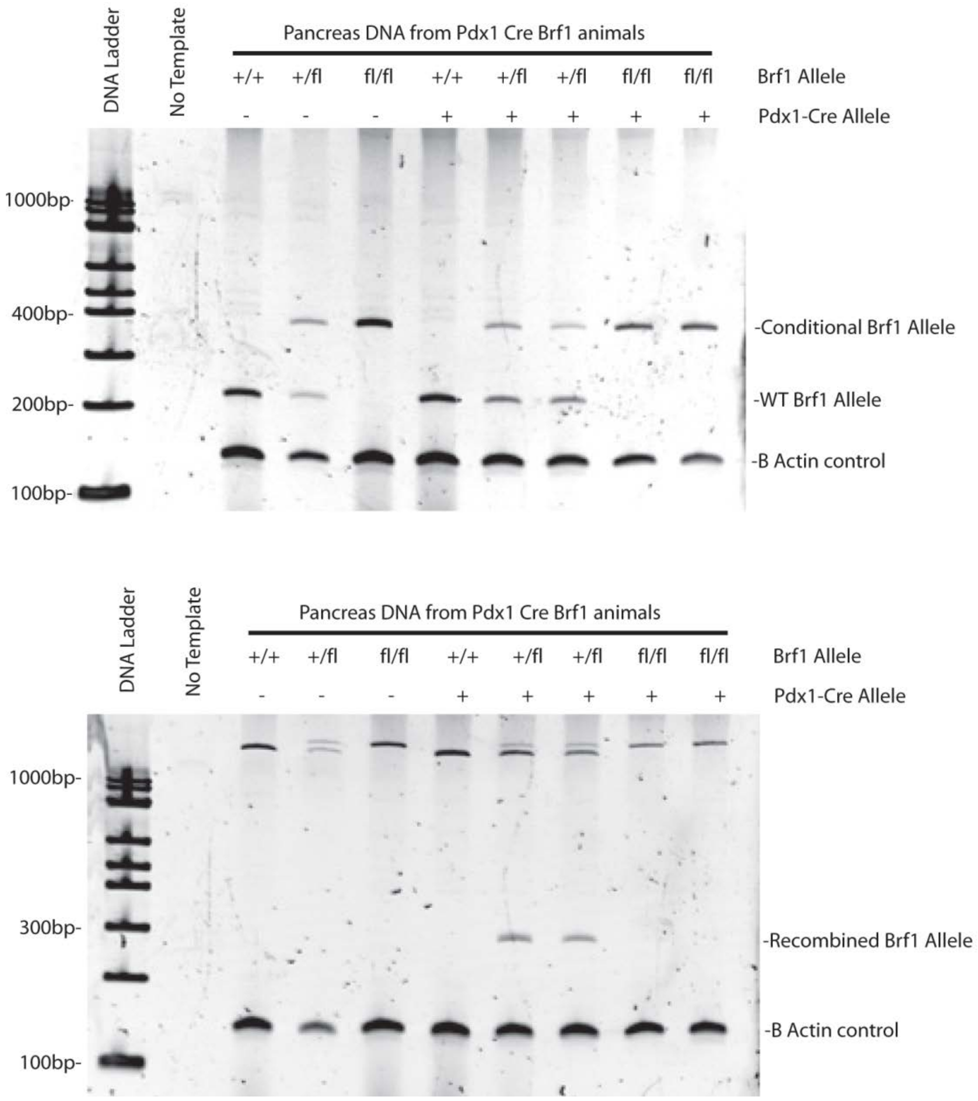

a)

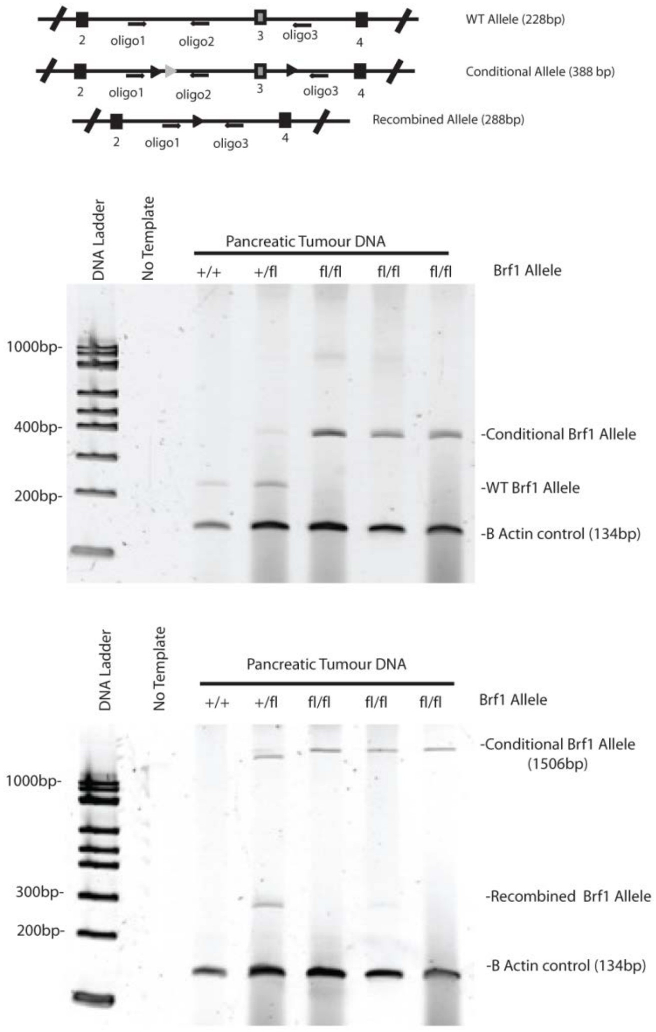
WT Allele (228bp)
Conditional Allele (388 bp)
Recombined Allele (288bp)
oligo1
oligo2
oligo3
DNA Ladder
No Template
Pancreatic Tumour DNA
+/+ +/fl fl/fl fl/fl fl/fl
Brf1 Allele
1000bp-
400bp-
200bp-
-Conditional Brf1 Allele
-WT Brf1 Allele
-B Actin control (134bp)
-Conditional Brf1 Allele (1506bp)
300bp-
-Recombined Brf1 Allele

b)

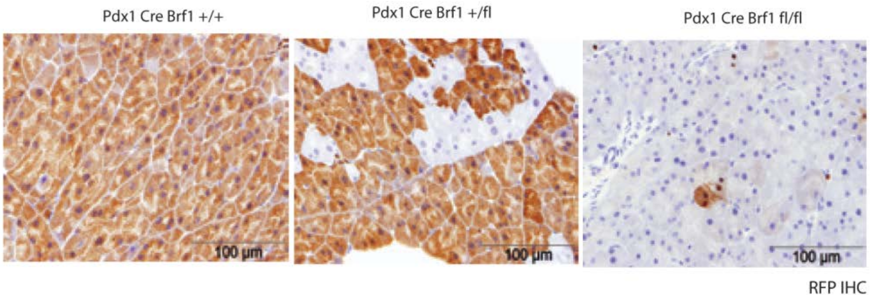
Pdx1 Cre Brf1 +/+
Pdx1 Cre Brf1 +/fl
Pdx1 Cre Brf1 fl/fl
100 µm
RFP IHC
