# Supplementary material for: Brf1 loss and not overexpression disrupts tissues homeostasis in the intestine, liver and pancreas
Source: Cell Death Differ. 2019 Mar 11;26(12):2535–50. doi: 10.1038/s41418-019-0316-7 (PMC6861133; doi:10.1038/s41418-019-0316-7)
Supplement: Supplementary file 2 — Supplemental tables [file 41418_2019_316_MOESM2_ESM.docx]

**Supplemental Table 1: Primers used**

| **Oligonucleotide Name** | **Oligonucleotide Sequence** |
| --- | --- |
| Oligo 1 | CCA GTT GTC CAG CCA AGA GG |
| Oligo 2 | CTCATATGCCATAAAACCCTATTCC |
| Oligo 3 | GTTGGAGGAAGTTTGTCATCAGG |
| Pre Oligo Forward | TCCTCTTCTGCCAAAAGTGG |
| Pre Oligo Reverse | CAGGTGCTTCTGCAGTCGTG |
| B-actin Forward | AGAGGGAAATCGTGCGTGAC |
| B- actin Reverse | CAATAGTGATGACCTGGCCGT |
| Pre B- actin Forward | CATTGTGATGGACTCCGGAGA |
| Pre B-actin Reverse | CAGACTTAACTTGTACTATGGC |
| Brf1 mRNA Forward | GCTGCAGCTAAACCAGCACT |
| Brf1 mRNA Reverse | GAACGTCTTCCCCAACACAT |
| tRNAiMET Forward | TGGTAGCAGAGGATGGTTTC |
| tRNAiMET Reverse | CTGGGCCCATAACCCAGAG |
| tRNAILE14 Forward | GGCGGCCGGTTAGCTCAG |
| tRNAILE14 Reverse | CCCTACGGGGATCGAAC |
| Mouse and Human Brf1 mRNA Forward | TGGCCCACGTGATTGCTGCC |
| Mouse and Human Brf1 mRNA Reverse | CAGCAGGTGCGCAAAGCGTG |
| U6 primers | Qiagen sequence: Cat number: QT02520980 Mm_LSM6_3_SG |

**Supplemental Table 2: Antibodies used for IHC**

| **Antibody for IHC** | **Company** | **Dilution** |
| --- | --- | --- |
| RFP | Rockland (600-401-379) | 1/100 |
| p53 (DO-7) | DAKO (M7001) | 1/1000 |
| p21 (M-19) | Santa Cruz (sc-471) | 1/800 |
| γH2AX | Cell Signalling (9718) | 1/50 |
| Cleaved Caspase 3 (ASP-175) | Cell Signalling (9661) | 1/50 |
| BRF1 | Bethyl Laboratories (270) | 1/150 |
| Pan-Cytokeratin | Thermo Scientific (MS-343) | 1/100 |
