# Supplementary material for: Brf1 loss and not overexpression disrupts tissues homeostasis in the intestine, liver and pancreas
Source: Cell Death Differ. 2019 Mar 11;26(12):2535–50. doi: 10.1038/s41418-019-0316-7 (PMC6861133; doi:10.1038/s41418-019-0316-7)
Supplement: Supplementary file 3 — Supplemental Figure legends [file 41418_2019_316_MOESM3_ESM.docx]

SUPPLEMENTAL FIGURE LEGENDS

S Fig 1: Confirming the cloning of the *Brf1^flox^* allele

a) Confirmation of 5’ site integration using two different restriction enzymes (*KpnI* and *Bsu36I*) and the depicted 5’ end probe. Southern blots are shown with the correct sizes for the specified genotypes.

b) Confirmation of 3’ site integration using the *BglI* restriction enzyme and the depicted 3’ end probe. A southern blot is shown with the correct sizes for the specified genotypes.

c) Confirmation of a single insertion site using the *SpeI* restriction enzyme and a probe that falls in the puro cassette (puro probe). A southern blot is shown with the correct sizes for the specified genotypes.

S Fig 2: Generating and assessing *Brf1* heterozygous mice

a) Diagram depicting inter-crossing of *Brf1^fl/fl^* mice with a ubiquitous *Deleter-Cre* mouse line (*Del-Cre*) to generate *Brf1^+/-^* animals.

b) Graph showing a weight comparison of *Brf1^+/+^* and *Brf1^+/-^* animals. Changes are not significant using a Mann-Whitney non-parametric test.

S Fig 3: Levels of γH2AX, cleaved caspase 3 and pan-cytokeratin upon loss of *Brf1*

a) IHC of liver samples from mice harvested at day 3, 4, 6 and 8 post-induction with an antibody against γH2AX. *Brf1*^+/+^ mice are a combination of Ah-CRE *Brf1*^+/+^ and mice without Ah-Cre while *Brf1*^fl/fl^ mice are Ah-Cre *Brf1*^fl/fl^ mice

b) IHC of liver samples from mice harvested at day 6 and day 8 post-induction with an antibody against cleaved caspase 3- (left) and an antibody recognising different cytokeratins- (right). *Brf1*^+/+^ mice are a combination of Ah-CRE *Brf1*^+/+^ and mice without Ah-Cre while *Brf1*^fl/fl^ mice are Ah-Cre *Brf1*^fl/fl^ mice

S Fig 4: Acute deletion of Brf1 in hepatocytes causes a liver collapse phenotype

a) A panel of IHC of sections taken from formalin fixed liver sampled from *Brf1^fl/fl^* mice induced with either AAV8-TBG-null vector (control virus) or AAV8-TBG-Cre (AAVCre) sampled Day 4, 6 or 8 post induction as indicated. As previously observed there was a change in morphology (H&E) and an increase in expression of p53, p21, caspase 3 and increase in pan-cytokeratin staining in the livers that had lost Brf1 from day 6 post induction. Scale bar = 50µm.

b) Serum samples taken from mice at the time points was analysed for specific liver enzymes as indicated. (Control virus n=4; AAVCre Day 4 n =3; AAVCre Day 6 n =3; AAVCre Day 8 n =5).

c) There was a decrease in the percentage liver weights of mice that had Brf1 loss at Day 8 post induction compared to uninduced wildtypes, (WT), control virus treated *Brf1^fl/fl^*. (WT n=3; Control virus n=4; AAV Day 8 n =8.)

d) RNA taken at day 8 after AAV induction was analysed by qPCR for tRNA^ILE14^ and U6 RNA expression. Wild-type expression is normalised to 1 and error bars represent SEM.

e) Whole liver lysates taken at day 10 (control virus and AAV Brf1^+/-^) or day 8 (AAV Brf1^-/-^) after AAV induction were analysed by western blotting for Brf1, using β-actin as a loading control.

S Fig 5: Human BRF1 rescues tRNA expression

a) Graph depicting levels of *BRF1* mRNA in the livers of mice, determined by qPCR with primers that recognise both human and mouse *BRF1* mRNA. Mouse genotypes are shown on the x-axis. Values are plotted relative to WT control. WT mice are *AhCre Brf1^+/+^*, Brf1 Hom mice are *AhCre Brf1^flfl^* and Brf1 Hom/TG mice are *AhCre Brf1^fl/fl^ BRF1^TG^* mice.

b) Graph depicting levels of tRNA^iMET^ and tRNA^ILE14^ in the livers of mice, determined by qPCR. Mouse genotypes are shown on the x-axis. Values are plotted relative to WT control for each tRNA. WT mice are *AhCre Brf1^+/+^*, Brf1 Hom mice are *AhCre Brf1^fl/fl^* and Brf1 Hom/TG mice are *AhCre Brf1^fl/fl^* HPRT^LSL-BRF1^ mice.

S Fig 6: Human BRF1 rescues the decrease in polysome loading.

Polysome profiles from liver samples of mice harvested at day8 post induction. *Brf1*^+/+^ mice are a combination of Ah-CRE *Brf1*^+/+^ and mice without Ah-Cre (n=3) while *Brf1*^fl/fl^ mice are Ah-Cre *Brf1*^fl/fl^ mice and Brf1^fl/fl^/LSL-BRF1 mice are Ah-CRE *Brf1*^fl/fl^ HPRT^LSL-BRF1^ mice containing an extra copy of human BRF1 (n=6).

S Fig 7: Human BRF1 expression does not induce proliferation of hepatocytes

a) Liver samples of control (*Brf1+/+*) and mice expressing a copy of human BRF1 (LSL-Brf1) after 8 days of β-naphthoflavone induction stained for H&E and with antibodies against BrdU and BRF1.

b) Quantification of BrdU positive hepatocytes. Numbers on the Y-axis represent the total number of BrdU positive cells after scoring 10 fields of 400x magnification. Panels are representative of at least three sacrificed mice per genotype. Brf1+/+, n-=5; LSL-Brf1, n=5.

c) Liver weights shown as fraction of body weight for WT mice (Brf1+/+, n=5) and mice expressing a human copy of Brf1 (LSL-BRF1, n=5) after 8 days of β-naphthoflavone induction. There is no significant difference when calculating p-value with a Mann-Whitney test.

S Fig 8: *Brf1* heterozygosity does not affect an intestinal tumorigenesis model

a) Kaplan Meier survival graph depicting intestinal tumor-free survival in *AhCre APC^fl/+^ Brf1^+/+^* mice (dark line, n=19) and *AhCre APC^fl/+^ Brf1^fl/+^* mice (red line, n=27).

b) Graphs showing tumor number (left), and tumor size (right), in *AhCre APC^fl/+^ Brf1^+/+^* (n=9) mice and *AhCre APC^fl/+^ Brf1^fl/+^* (n=8) mice. A Mann-Whitney test was used to calculate p-values for both graphs.

c) qPCRs from RNA isolated from mouse livers 10 days after AAV induction. Brf1fl/- mice were treated with control virus or AAV-TBG-Cre and sampled 10 days later.

S Fig 9: Deletion of Brf1 is detrimental to developing pancreas

PCR blots showing recombination at the *Brf1* locus in Pdx1-Cre Brf1^fl/fl^ mice. A depiction of primers used and PCR products is shown in Figure 2D. Top: PCR products using oligo 1/oligo 2 primer combinations and DNA isolated from pancreata of the animals of the genotype shown above each lane. Bottom: PCR products using oligo 1/oligo 3 primer combinations and DNA isolated from pancreata of the animals with genotypes shown above each lane. Notice in this case the presence of only the conditional allele (at 1506bp) in *Pdx1-Cre Brf1^fl/fl^* derived tumours. β-actin was used as control in the same PCR.

S Fig 10: Confirming heterozygous deletion of Brf1.

a) Gel electrophoresis of PCR products showing recombination at the *Brf1* locus in KPC *Brf1^fl/fl^* mice. The schematic shows the genotyping strategy and size of PCR products for the WT, conditional (*Brf1^flox^*), and recombined (*Brf1^-^*) alleles. The top gel shows PCR for the conditional vs wild-type *Brf1* alleles in DNA isolated from KPC tumours with the indicated *Brf1* genotype. The bottom gel shows PCR for the conditional (non-recombined) and recombined *Brf1* allele in DNA isolated from 5 KPC tumours with the indicated *Brf1* genotype. Note the conditional allele (at 1506bp) is retained in KPC *Brf1^fl/fl^* derived tumours. β-actin was used as a control in the same PCR.

b) Immunohistochemistry for RFP on sections of formalin-fixed paraffin embedded (FFPE) pancreatic tissue from mice of the genotypes indicated
